# Human Urine-Derived Stem Cells Improve Partial Bladder Outlet Obstruction in Rats: Preliminary Data and microRNA-mRNA Expression Profile

**DOI:** 10.1007/s12015-022-10340-0

**Published:** 2022-03-01

**Authors:** Menjiang Tu, Rui Wang, Pei Zhu, Qingqing Wang, Bishao Sun, Keshi Lu, Jiawei Zhang, Weijie Xie, Huan Guo, Shulin Li, Yuqi Wu, Xiangwei Wang

**Affiliations:** 1 Department of Urology, Southern University of Science and Technology Hospital, Liuxian Street, Nanshan District, Shenzhen, NO.6019 China; 2 Department of Urology, Second Affiliated Hospital, Third Military Medical University, Chongqing, China

**Keywords:** partial bladder outlet obstruction, human urine-derived stem cells, gene expression profiles, microRNA, miR-142, miR-9a

## Abstract

Partial bladder outlet obstruction (pBOO) often results in bladder tissue inflammation and remodeling. As human urine-derived stem cells (USCs) have demonstrated therapeutic benefits, we used a rat model to investigate the effect of USCs on bladder function and explore the miRNA and gene expression profiles in bladder tissue using RNA sequencing. Eighteen rats were assigned to a sham surgery group, pBOO group, and pBOO+USC group (six biweekly treatments). Routine urodynamic monitoring, analysis of detrusor muscle strips, and pathophysiology assessments were conducted. Finally, altered miRNA and mRNA expression profiles of bladder tissue were examined using RNA sequencing and bioinformatics analysis. After USC treatment, elevated bladder compliance and maximal voiding pressure, declined end filling pressure and voided volume, and improved detrusor muscle contractility and carbachol sensitivity were found. Histology and TUNEL assay revealed reduced collagen deposition and muscle cell apoptosis in bladder tissue. The differential expression of eight miRNAs was reversed by USC treatment. Two large nodes (miR-142 and miR-9a) were identified in the miRNA-gene interaction network in the USC-treated group. The Kyoto Encyclopedia of Genes and Genomes analysis revealed enrichment of multiple significant pathways, including those involved in necroptosis and cytokine-cytokine receptor interactions. This is the first study to demonstrate the protective effect of USCs on bladder function and remodeling in pBOO rats. The miRNA and mRNA expression levels differed in the bladder of pBOO rats with and without USC treatment. Although the mechanism underlying these effects has not been fully elucidated, necroptosis and cytokine-cytokine receptor interaction-related pathways may be involved.

**Graphical abstract:**

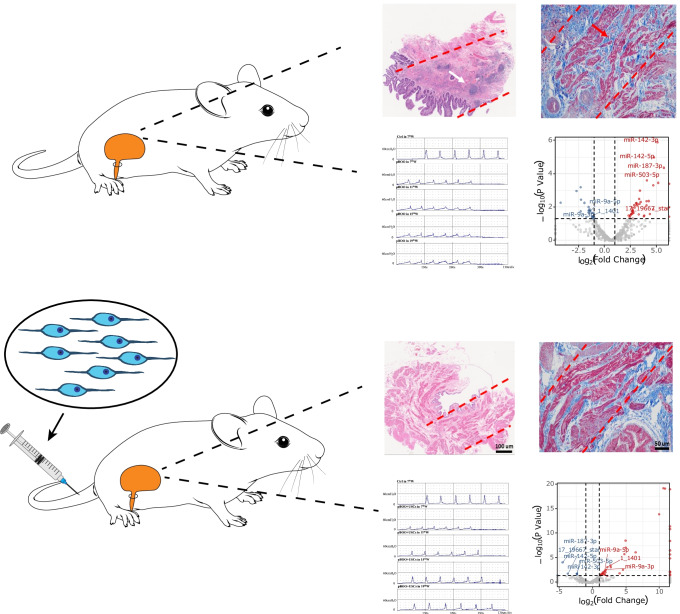

**Supplementary Information:**

The online version contains supplementary material available at 10.1007/s12015-022-10340-0.

## Background

Partial bladder outlet obstruction (pBOO) is defined as a common urological disorder in which a partial obstruction of any part between the bladder outlet and external urethra is observed. The most common causes of pBOO are benign prostate hyperplasia in elderly men, posterior urethral valves in newborns, pelvic prolapse in women, and urethral stricture caused by iatrogenic injury. In patients with moderate or severe long-term pBOO, an increase in bladder outlet resistance pressure and intravesical pressure results in bladder tissue inflammation, detrusor muscle hypertrophy, connective tissue accumulation in the bladder wall, and tissue remodeling and decreased bladder compliance [1]. Even after the obstruction is relieved, bladder dysfunction can persist. Symptoms such as waiting for urine, weak urination, frequent urination, and nocturia may remain or even worsen.

Owing to their self-renewal ability, multidirectional differentiation, and proliferation, stem cells have demonstrated tissue repair capabilities during the remodeling and fibrosis in multiple organ systems. Studies have confirmed that mesenchymal stem cells can inhibit the inflammatory response and fibrosis process of bladder tissue after the development of pBOO, thereby improving bladder function [2]. Urine-derived stem cells (USCs) are recognized as pluripotent stem cells with multidirectional differentiation potential. USCs can be directly extracted from urine more easily than extracting mesenchymal stem cells, and the process is therefore convenient and economical and does not require invasive surgery. For patients without kidney disease, USCs can be extracted directly from the urine, and this demonstrates a lower risk of occurrence of autoimmune reactions and involves minor ethical issues.

In this study, we used human USCs in a pBOO rat model with an aim to clarify the effect of USCs on bladder function by observing bladder urodynamics and histological changes and investigated the microRNA (miRNA) and mRNA expression profiles of bladder smooth muscle tissue using high-throughput sequencing. We used bioinformatics methods to clarify the potential molecular pathways of USCs acting on bladder tissue.

## Methods

### Ethics Statement

The experiments were approved by the Animal Care and Use Committee of Shenzhen University General Hospital and complied with the Guide for the Care and Use of Laboratory Animals, Eighth Edition (2011). Written informed consent was obtained from all donors prior to the collection of urine samples.

### Animals and Study Design

The eighteen adult male Sprague-Dawley rats used in this study were purchased from the Center for Experimental Animals of Guangzhou University of Chinese Medicine. All rats weighed 190–210 g and were housed at 20–26 °C under a standard 12 h/12 h light-dark cycle. The rats were randomly and evenly assigned to the following three groups: a sham surgery group (control group), a pBOO without USC therapy group (pBOO group), and pBOO with USC therapy group (pBOO+USCs group).

### Induction of the pBOO Model

The rats in the pBOO and pBOO+USCs groups were subjected to urethral ligation under anesthesia using isoflurane to establish the pBOO model, as per methods described previously [3]. Briefly, with the rat placed in the supine position, a midline suprapubic incision was inflicted, and the bladder neck and urethra were exposed. A 1.0-mm inner diameter catheter was placed around the proximal urethra, and a 4-0 silk ligature was tied around the urethra and tube. After checking for tightness, the tube was removed, and the incision was closed. Six rats in the control group were subjected to the same operation to expose the bladder without urethral ligation.

### Isolation and Culture of USCs

USCs were collected from the urine of five healthy male donors aged 19–25 years. The collection, isolation, and identification methods have been described in our previous study [4]. Briefly, urine samples were centrifuged at 400× *g* for 5 min to collect the cells. The cell pellet was gently resuspended in a mixed medium consisting of embryo fibroblast medium and keratinocyte serum-free medium (1:1 ratio), following which the cells were seeded in 24-well plates (passage (P) 0). Individual USCs were observed 3–5 days after the initial seeding and reached 70%–80% confluency after another 3–4 days. The cells were trypsinized and transferred to 6-well plates (P1). Finally, the cells were transferred to a 100-mm culture dish (P2) for expansion. P3 USCs were used in the experiments.

### Treatment with USCs

The pBOO model was successfully established via urodynamic analysis seven weeks after performance of the urethral ligation procedure. Hence, all rats in the pBOO+USCs group were administered with a tail vein injection of USCs (2× 10^6^ cells suspended in 0.2 mL phosphate-buffered saline [PBS]) six times every other week. Rats in the pBOO group received an injection of an equal volume of PBS using the same process.

### Urodynamics

Urodynamic studies were performed at the 7^th^ week post-urethral ligation to determine the baseline urodynamic values, and urethral ligation was released for rats in the pBOO groups. All rats were subjected to urodynamic studies 7, 11, 15, and 19 weeks after urethral ligation. Under subjection to isoflurane anesthesia through an abdominal incision, a 27-gauge double lumen catheter was inserted into the bladder and connected to a pressure transducer (MLT488, ADinstruments, Australia). The bladder was emptied using a syringe and the abdomen was covered with a warm gauze. Saline solution was infused through the catheter at a constant rate of 0.3 mL/min. The following parameters were recorded: end-filling pressure, voided volume, maximal voiding pressure, bladder capacity, and residual volume. Bladder volume change (ΔV) was calculated as the perfusion volume from infusion to urine leakage, and bladder pressure change (ΔP) was calculated as the bladder leak point pressure minus the pressure before bladder perfusion. Bladder compliance (compliance, C) was calculated using the formula ΔV/ΔP.

### Measurement of Bladder Muscle Strip Tension

At the end of the experiments, all rats were euthanized via subjection to an isoflurane anesthetic overdose. The bladder was rapidly removed and subjected to fixation in Krebs–Henseleit solution at 4 °C. The mucosa was carefully removed under a microscope and excised into longitudinal muscle strips (8 ×2 mm) using a double-edged knife. All muscle strips were transferred to a 10-mL organ bath filled with Krebs–Henseleit solution. The bath was maintained at 37 °C under an 95% O_2_ and 5% CO_2_ atmosphere. After warming for 30 min without tension, one end of each strip was ligated to a force transducer (MLT488, ADinstruments, Australia), and the other end was fixed to the bottom of the fixing plate with a 4-0 silk ligature. The force transducer-linked tension sensor was connected to a computer to detect and record the signal using the Powerlab multichannel biological signal analysis system (MI224, ADinstruments, Australia). The fine-tuning spiral was adjusted to slowly pull the muscle strip to increase the tension of the muscle strip. Contraction was quantified by measuring the force generated in millinewton (mN) (g = 9.81). The basic value of tension was 0.7 mN, which was maintained for 15 min. After the muscle strips were balanced, 10^-4^ M carbachol was added to stimulate the muscle strips, and the maximum contraction value of the muscle strips was recorded.

### Histological Examination

Half of the bladder was snap-frozen in liquid nitrogen for the conduction of microarray expression profiling and bioinformatics analysis. The remaining tissues were subjected to fixation in 4% paraformaldehyde and stained paraffin-embedded before analysis. The sections were stained with hematoxylin-eosin (H&E) to observe the general morphology of the bladder, and Masson’s trichrome staining was performed to evaluate the extent of tissue fibrosis. All sections were evaluated under a light microscope (Olympus, Tokyo, Japan).

### TUNEL Assay

A one-step TUNEL apoptosis assay kit (Beyotime Biotechnology, Shanghai, China) was used to evaluate apoptosis levels in the bladder smooth muscle. Briefly, the sections were regularly hydrated and immersed in citric acid buffer (0.01 mM, pH = 6), followed by incubation with 10% bovine serum albumin for 30 min. Consequently, the sections were treated with the TUNEL reaction solution for 20 min and then with DAPI for 10 min in a dark chamber. Finally, after regular dehydration and transparency, the sections were observed under a fluorescence microscope (Olympus, Tokyo, Japan) and analyzed by a pathologist in a blinded manner.

### RNA Sequencing

At the end of the experiment, total RNA extraction was performed from a section of the bladder tissue obtained from two rats in the control group, two rats in the pBOO group, and four rats in the pBOO+USCs group, and the samples were processed for RNA sequencing. A total of 1 μg RNA per sample was used as the input material for the RNA sample preparation. Sequencing libraries were generated using the rRNA-depleted RNA and NEBNext® Ultra™ Directional RNA Library Prep Kit for Illumina® (NEB, USA), following the manufacturer’s recommendations. The library integrity was assessed using the Agilent Bioanalyzer 2100 system (Agilent, USA). Clustering of the index-coded samples was performed using the cBot Cluster Generation System and TruSeq PE Cluster Kit v3-cBot-HS (Illumina), according to the manufacturer’s instructions. After the completion of the cluster generation, the libraries were sequenced using the Illumina Hiseq 3000 platform, and 150-bp paired-end reads were generated. Reference genome and gene model annotation files were downloaded directly from the Genome website. The mapped reads of each sample were assembled using both Scripture (beta2) and Cufflinks (v2.1.1). Raw read counts were then used as input to DESeq to calculate the normalized signal for each transcript in the samples. The *p* value was adjusted using the q value. A q value <0.01 and |log_2_(fold change)| >1 were set as the thresholds for significantly differential expression. For small RNA sequencing, mapping was performed using the reference sequence and Bowtie [5]. miRBase20.0 was used as a reference (6). The target gene of miRNA was predicted using psRobot_tar in miRanda for animals [7]. In the present study, FUNRICH (http://www.funrich.org/) was used to perform Gene Ontology (GO), while the Kyoto Encyclopedia of Genes and Genomes (KEGG) was used for pathway analyses.

### Statistical Analysis

All values are presented as mean±SD. Statistical analyses were performed using SPSS v.22.0 (SPSS Inc., Chicago, IL, USA) and Graphpad Prism v.8.0 (GraphPad Software, San Diego, CA, USA). The Two-way ANOVA and One-way ANOVA analysis of variance was performed to evaluate the differences among the rat groups considered herein. Statistical significance was set at *p* <0.05.

## Results

### Urodynamics

**Fig. 1:**
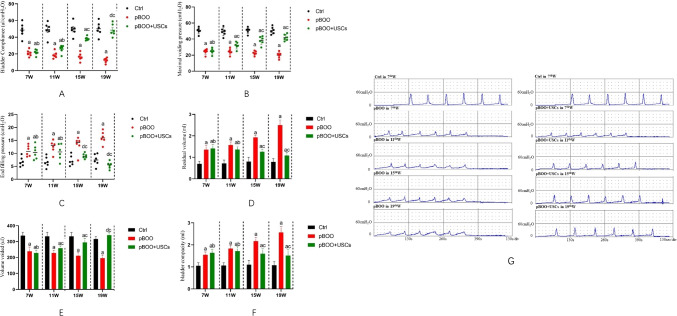
Effects of urine-derived stem cells (USCs) on bladder function. (**A**–**F**) Cystometric parameters, end-filling pressure, residual volume, and bladder capacity increased, and maximal voiding pressure, voided volume, and bladder compliance decreased in partial bladder outlet obstruction (pBOO) rats. Injection with USCs improved bladder micturition function with respect to end-filling pressure, maximal voiding pressure, and voided volume, thereby resulting in an increase in bladder compliance. a: *p* <0.05, *vs.* control group, b: *ns*, *vs.* pBOO group, c: *p* <0.05, *vs.* pBOO group, d: *ns*, *vs.* control group. (**G**) Representative cystometrograms depicted for three rat groups at 7, 11, 15, and 19 weeks after urethral ligation

Urodynamic studies in all groups revealed the highest bladder compliance in controls and the lowest compliance in both pBOO groups in the 7^th^ week post-urethral ligation (48.57±8.61 *vs.* 21.00±3.87 μL/cm H_2_O, respectively, *p* < 0.01). After being subjected to two injections of USCs, compliance gradually improved in the pBOO+USCs group in the 15^th^ week compared with that in the pBOO group (38.35±2.81 *vs.* 16.57±4.69 μL/cm H_2_O, respectively, *p* < 0.01) and was similar to that of the controls in the 19^th^ week (48.50±7.28 *vs.* 50.07±8.04 μL/cm H_2_O, respectively, *p* = 0.89) (Fig. 1A). The maximal voiding pressure in the pBOO group was significantly lower than that in the control group in the 7^th^ week (24.32±3.22 *vs.* 46.80±10.17 cm H_2_O, respectively, *p* < 0.01), whereas pBOO+USCs gradually presented with an increasing pressure from the 15^th^ week (38.31±5.21 *vs.* 22.61±2.60 cm H_2_O, respectively, *p* < 0.01) (Fig. 1B). End-filling pressure in pBOO rats was higher than that in controls (10.96±2.07 *vs.* 6.84±1.80 cm H_2_O, respectively, *p* = 0.006), and it increased from the 7^th^ to 19^th^ week (10.96±2.07 in 7^th^ week *vs.* 16.15±1.68 cm H_2_O in 19^th^ week, *p* < 0.01); however, the pressure in pBOO+USCs rats decreased to the control level (9.03±0.99 *vs.* 7.26±1.94 cm H_2_O, respectively, *p* = 0.359) in the 15^th^ week (Fig. 1C). All pBOO and pBOO+USCs rats demonstrated significantly higher residual volume than that in control rats (0.70±0.11 *vs.* 1.36±0.19 mL, respectively, in the 7^th^ week [*p* < 0.01]); however, USCs considerably suppressed the upward trend from the 15^th^ week (1.26±0.12 *vs.* 1.93±0.10 mL, *p* < 0.01) (Fig. 1D). Moreover, pBOO rats exhibited lower voided volume than that in control rats in the 7^th^ week (238.77±27.11 *vs.* 337.84±21.49 μL, *p* < 0.01); however, the pBOO+USCs group exhibited an increasing trend and finally demonstrated similar voided volume as the control group in the 19^th^ week (340.75±16.93 *vs.* 316.51±16.23 μL, respectively, *p* = 0.119) (Fig. 1E). Additionally, a significant increase in the bladder capacity was observed in all pBOO rats compared with that in the control (1.54±0.14 *vs.* 1.04±0.14 mL, respectively, *p* < 0.01). Although USCs reversed the trend in the pBOO+USCs group from the 15^th^ week (1.59±0.23 *vs.* 2.17±0.17 mL, *p* < 0.01), the bladder capacity did not decrease to the level of the control (1.52±0.27 *vs.* 1.08±0.16 mL, respectively, *p* < 0.01) (Fig. 1F). Representative cystometrograms of three rat groups at 7, 11, 15, and 19 weeks after urethral ligation are presented in Fig. 1G.

### Histology and TUNEL Assay

**Fig. 2:**
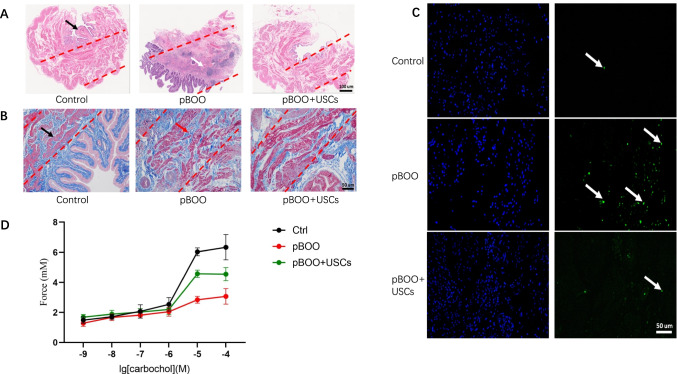
Changes of bladder wall histology and detrusor muscle contraction. (**A**–**C**) Hematoxylin and eosin staining, Masson's trichrome staining, and TUNEL assay revealed the deposition of a fibrous matrix and cellular apoptosis in the bladder wall, and USC treatment improved the fibrosis and cell apoptosis in the bladder wall of pBOO+USCs rats. (**D**) The detrusor muscle contractility, induced by carbachol, was significantly lower in pBOO rats than that in control rats. The pBOO+USCs rats demonstrated a higher sensitivity to carbachol in detrusor muscle contraction

H&E staining revealed that the bladder wall thickness is higher, the urothelium flatter, and structural damage of detrusor smooth muscle worse in the pBOO group than those observed in the control group; however, treatment with USCs significantly alleviated these histological changes (Fig. 2A). Masson’s trichrome staining revealed the presence of increased collagen content, disorderly and loosely arranged detrusor muscle bundles, and an evidently widened gap between muscle bundles in the pBOO group compared with that in the control group and pBOO+USCs group (Fig. 2B). There was a marked increase in the number of TUNEL-positive cells in the pBOO group compared with that in the control group (48.17±7.38 *vs.* 5±1.29, respectively, *p* < 0.01), whereas USC treatment significantly decreased the number of apoptotic cells in the bladder (32.17±5.52 *vs.* 48±7.38, respectively, *p* < 0.01) (Fig. 2C).

### Assessment of Detrusor Muscle Contractility

In the detrusor tension tests, the bladder detrusor contraction amplitudes of pBOO rats were significantly lower than those of control rats. Carbochol elevated the contraction amplitudes at different concentrations (10^-6^ to 10^-4^ M) in the three groups, but the bladder detrusor of pBOO rats exhibited the lowest sensitivity to carbochol. The effect of carbochol on the amplitudes of contraction in pBOO+USCs rats was significantly higher than that in pBOO rats (4.58±0.89 *vs.* 2.85±0.23 mM, respectively, *p* < 0.01); however, it was lower than that in control rats (4.58±0.89 *vs.* 6.04±0.26 mM, respectively, *p* < 0.01) (Fig. 2D).

### Bioinformatics Analysis of miRNA–mRNA

Bioinformatics analysis revealed differentially expressed genes (DEGs) and differentially expressed miRNAs (DEMs) in the bladder wall tissue among the control, pBOO, and pBOO+USCs groups in rats. Below, we have provided the results for miRNAs and mRNAs that were regulated in the pBOO and USC treatment relative to the controls. The comparisons are therefore pBOO relative to the control group and pBOO+USCs relative to the pBOO group.

**Fig. 3:**
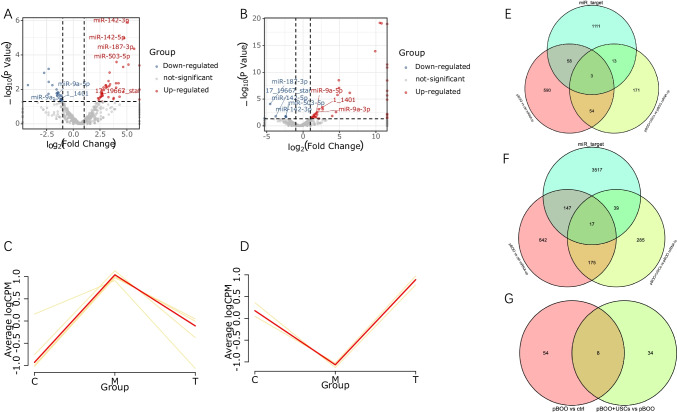
Differentially expressed miRNAs (DEMs) and differentially expressed genes (DEGs) changes induced by pBOO and USCs. Volcano plots illustrating DEMs in rats in the pBOO *vs.* control group (**A**) and the pBOO+USCs *vs.* pBOO group (**B**). Log_2_ transformation value of the fold change in miRNA expression between each group plotted on the x-axis. The log p value (base 10) is plotted on the y-axis. DEMs (fold change ≥1) are indicated in red (upregulated miRNAs) and blue (downregulated miRNAs). (**C**–**D**) The gene trend of DEMs in the three groups. (**E**) The overlapping target genes of the five DEMs and downregulated mRNAs expressed in rats from pBOO *vs.* control group (pBOO *vs.* ctrl mRNA_lo) and upregulated mRNAs expressed in rats from pBOO+USCs *vs.* pBOO group (pBOO+USCs *vs.* pBOO mRNA_up) were identified using Venn diagrams. (**F**) The overlapping target genes of the five DEMs and upregulated mRNAs expressed in rats from pBOO *vs.* control group (pBOO *vs.* ctrl mRNA_up) and downregulated mRNAs expressed in rats from pBOO+USCs *vs.* pBOO group (pBOO+USCs *vs.* pBOO mRNA_lo) were identified using Venn diagrams

The volcano plots in Figure 3a and b show DEMs in rat bladder wall tissues of the control, pBOO, and pBOO+USCs groups. The distribution of all miRNAs with respect to significance (y-axis) *vs.* foldchange (x-axis) has been illustrated using the volcano plot. The present study identified 62 DEMs (40 upregulated and 22 downregulated) and 1686 DEGs (981 upregulated and 705 downregulated) in the pBOO group compared with the control group. Furthermore, we identified 42 DEMs (25 upregulated and 17 downregulated) and 757 DEGs (241 upregulated and 516 downregulated) in the pBOO+USCs group compared with the pBOO group. The expression of eight DEMs, which were upregulated in pBOO rats relative to control rats, was reversed via USC treatment (Fig. 3).

Five of the eight DEMs, namely miR-142-3p, miR-142-5p, miR-187-3p, miR-503-5p, and 17_19667_star, presented upregulated expression in pBOO rats (Fig. 3A) and the expression level was downregulated by USC treatment (Fig. 3B). The Venn diagrams illustrating downregulated mRNAs expressed in rats from pBOO *vs.* control group (pBOO vs. ctrl mRNA_lo) and upregulated mRNAs expressed in rats from pBOO+USCs vs. pBOO group (pBOO+USCs vs. pBOO mRNA_up), and the target genes of the above-mentioned five DEMs, helped identify three DEGs (Fig. 3).

Three of the eight DEMs, namely miR-9a-3p, miR-9a-5p, and 1_1401, exhibited downregulated expression in pBOO rats (Fig. 3A) and upregulated expression via USC treatment (Fig. 3B). Similarly, the Venn diagrams illustrating upregulated mRNAs expressed in rats from pBOO *vs.* control group (pBOO *vs.* ctrl mRNA_up) and downregulated mRNAs expressed in rats from pBOO+USCs *vs.* pBOO group (pBOO+USCs *vs.* pBOO mRNA_lo), and the target genes of the above-mentioned three DEMs, helped identify 17 DEGs (Fig. 3). The changes in the levels of these DEMs in the three groups are shown in the gene trend map in Figure 3C and D.

**Fig. 4:**
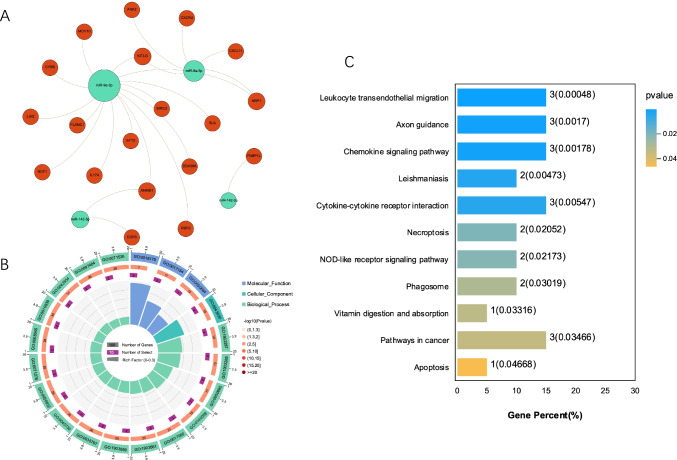
DEMs-DEGs regulatory network and bioinformatic analysis of bladder miRNA-mRNA. (**A**) Regulation networks of miRNA–mRNA were constructed using the Gephi software (version 0.9.1). Green dots represent miRNAs, whereas red dots indicate downstream target genes. (**B**) Enriched Gene Ontology (GO) terms for target genes. (**C**) Kyoto Encyclopedia of Genes and Genomes (KEGG) analysis of the genes in the regulation networks of miRNA–mRNA

Following the above-mentioned analysis, a miRNA–gene interaction network was constructed by integrating data on the target genes with DEGs. As shown in Figure 4A, the pBOO and USC-associated DEM–DEG interaction network was constructed, which comprised 23 nodes, including four that were DEMs, and 22 connections. miR-9a-3p and miR-9a-5p were determined as the two largest nodes of the four DEMs and established interactions with 13 and five DEGs, respectively.

GO analysis revealed that the most significantly enriched GO terms that correspond to the above-mentioned target DEGs were “superoxide-generating NADPH oxidase activity” (ontology: MF), “NADPH oxidase complex” (ontology: CC), and “semaphorin-plexin signaling pathway involved in axon guidance” (ontology: BP) (Fig. 4B).

Furthermore, KEGG analysis revealed that multiple significant pathways were enriched, which were primarily associated with leukocyte transendothelial migration, chemokine signaling pathway, cytokine–cytokine receptor interaction, necroptosis, nucleotide-binding and oligomerization domain (NOD)-like receptor signaling pathway, and apoptosis signaling pathway (Fig. 4C).

## Discussion

PBOO initiates a pathophysiological cascade of events in the bladder wall, including inflammation, hypoxia, collagen deposition, smooth muscle hypertrophy, apoptosis, and fibrosis. The obstructed bladder undergoes modification with respect to its structure to compensate for the increased resistance to flow, and significant hypoxia ensues owing to the development of high resistance to flow and consequent high intravesical pressure [8,9]. Studies in 2 week-obstructed rabbit models revealed the existence of a larger capacity and lower intravesical pressure and compliance of the bladder [8]. Additionally, studies in Sprague-Dawley rats have shown an increase in end-filling pressure, residual volume, and bladder capacity, as well as a decrease in maximal voiding pressure and bladder compliance [3,10]. Some parameters such as voided volume vary with the duration of obstruction time. Yuan et al. revealed that voided volume increased in 3 week-obstructed rats and decreased in the 6 week-obstructed group [9]. Bladder strip contractility tension and tension sensitivity reportedly decrease in various animal models, including mice, rats, and rabbits [3,8,11].

Presently, effective treatments for the improvement of bladder function after long-term moderate to severe pBOO are unavailable in clinical practice. Studies have confirmed the effect of MSCs on the bladder function of pBOO animal models based on the potential applicability of stem cells in tissue reconstruction and organ repair engineering [8, 12–14]. However, the mechanism has not been explored at the genetic and molecular levels. Additionally, the disadvantages of MSCs, including the invasive extraction process and high cost, have limited their future clinical applications. Compared with MSCs, USCs are deemed advantageous owing to the simple, noninvasive, and low-cost harvesting methods. Studies have reported that the nephron-protective effect of USCs on renal function is realized via anti-inflammatory, antioxidative stress, and antifibrotic activities in acute and chronic kidney disease [15,16]. Moreover, USC-exosomes exhibit pro-neurogenesis effects in the rat brain after the occurrence of ischemic stroke and exert of pro-angiogenesis effects and improve erectile dysfunction in diabetic rats, thereby exhibiting bladder-protective functions [17–19]. Nonetheless, there are no reports available on the effects of USCs on the bladder function in patients with pBOO.

To explore the effect of USCs on bladder function and morphology in moderate or severe pBOO, we designed a long-term pBOO rat model and dynamically observed the changes from 7- to 19-week-obstructed rats. Similar to the findings reported in previous studies, we observed elevated end-filling pressure, residual volume, and bladder capacity and declined maximal voiding pressure, voided volume, and bladder compliance in the pBOO group compared with those in the control group. Additionally, once a low-compliance bladder was established, cystometric parameters did not improve without intervention, even if the urethral ligature was removed. Certain parameters, including end-filling pressure, residual volume, and bladder capacity, consistently increased after ligature removal, which consequently resulted in worsening of bladder function. Similar to that observed in most patients with moderate to severe long-term prostatic hyperplasia, the lower urinary tract symptoms of patients with pBOO could not be relieved even if there is acceptance of resection of the prostate to relieve bladder outlet obstruction. After subjection to treatment with USCs, few cystometric parameters were significantly improved, including elevated bladder compliance and maximal voiding pressure, declined end-filling pressure, and voided volume. Although residual volume and bladder capacity did not show recovery, there was no further deterioration after subjection to USC treatment compared with the pBOO group. The detrusor muscle contractility and carbachol sensitivity were also improved after USC treatment. Results from the histological analysis and TUNEL assay revealed that the improvement of these functions was mainly related to the reduction of collagen deposition in the extracellular matrix and to the reduction of muscle cell apoptosis. However, this study did not explore the mechanism underlying the improving effect of USCs on bladder function and morphology in moderate or severe pBOO. The intravenous route lacks reliably good uptake into the target tissue because stem cells in the venous system can be trapped in the lung capillaries. Our previous study showed that fibrosis and apoptosis of the myocardium, glomerulus, and detrusor were significantly inhibited by the injection of USCs via tail veins in a rat model of diabetes mellitus, although injected USCs were only observed in the pancreas and kidney, but not in the heart and bladder [19]. Some studies have also revealed the therapeutic effects of different stem cells injected through the tail vein on several injured organs in rat models [2, 15]. USCs may not exert their therapeutic actions only by homing into damaged target organs and differentiating there into functional cells. USC-exosomes have been extensively studied in recent years, and they can promote renal injury repair and angiogenesis [20,21]. Therefore, USCs might act by secreting exosomes to regulate the physiological and pathological process in pBOO rat model, which needs further study.

Furthermore, the urinary bladder was exposed to mechanical stress and was subjected to treatment with USCs, which undoubtedly resulted in modification of gene and protein expression profiles in epithelial and smooth muscle cells, consequently altering the ultrastructure and physiology of cells in the bladder wall. To elucidate the molecular mechanisms underlying this complex process, we conducted bioinformatics analysis of miRNA–mRNA expression profiles of rats in the three groups. We focused on the numerous miRNAs and mRNAs that were significantly and differentially expressed in the bladder following pBOO; those that underwent reversed expression following USC treatment were examined. We identified miR-142 and miR-9a as the two largest nodes of DEMs in the miRNA–gene interaction network. MiR-142 and miR-9a have the same gene sequence in humans and rats, indicating that miR-142 and miR-9a are conserved sequences. Therefore, we believe that the role of miR-142 and miR-9a in the rat model is similar to that in humans.

MiR-142-3p and miR-142-5p are reportedly overexpressed in the bladder tissue of pBOO mice [22]. A previous study confirmed that miR-142-5p is overexpressed in the bladder of patients with pBOO [23]. MiR-142-3p and miR-142-5p influence inflammation and immune response, the levels of which have been shown to decrease in mouse models of cardiac hypertrophy. miR-142-5p targets acetyltransferase p300 and is implicated in cardiac growth; additionally, an increase in miR-142 expression during cardiac growth is critical for cell survival [24,25]. The *in vivo* inhibition of miR-142-5p with locked nucleic acid-modified oligonucleotides reduces CCL4-induced liver fibrosis and bleomycin-induced lung fibrosis in mice. Furthermore, macrophages derived from the tissue samples of patients with liver cirrhosis and idiopathic pulmonary fibrosis display increased levels of miR-142-5p [26]. Therefore, it can be inferred that miR-142-5p regulates macrophage profibrogenic gene expression during chronic inflammation. In this study, miR-142-3p and miR-142-5p were overexpressed in pBOO rats, but their expression was suppressed by USCs, indicating that miR-142 participated in the antifibrosis effect of USCs in pBOO rats.

Studies have revealed that miR-9a participates in anti-inflammatory and anti-apoptotic functions and is related to neurogenesis. The downregulation of miR-9a-5p expression may increase the levels of NLRP1 inflammasome proteins, cleaved caspase-1, interleukin (IL)-1 b, and IL-18. In contrast, the overexpression of miR-9a-5p markedly prevents the abnormal expression of these proteins and is accompanied by the downregulation of NLRP1 expression in ischemia-like conditions, both *in vivo* and *in vitro* [27]. Further, miR-9 prevents cardiac dysfunction and inhibits cardiomyocyte apoptosis in myocardial infarction mouse hearts [28]. However, a study indicated that miR-9a-5p is a novel regulator of HSCs in the setting of increased pressure and hepatic fibrosis in rats and that the overexpression of miR-9a-5p following hepatic stellate cell activation perpetuates the fibrotic response [29]. In our study, we found that both miR-9a-3p and miR-9a-5p levels were decreased in pBOO rats and increased in USC-treated pBOO rats. They may protect bladder function by reducing the inflammatory response of bladder tissue and reducing detrusor cell apoptosis, aspects which should be confirmed through further studies.

To obtain insights into the classification of miRNAs, we conducted GO and KEGG analyses to predict the target genes and possible pathways. The results revealed various GO terms associated with superoxide-generating NADPH oxidase activity, NADPH oxidase complex, semaphorin receptor activity, semaphorin–plexin signaling pathway involved in axon guidance, oxidoreductase activity, acting on NAD(P) H, oxygen as an acceptor, and hydrogen peroxide biosynthetic process. Additionally, it was observed that various pathways were involved, including leukocyte transendothelial migration, chemokine signaling pathway, cytokine–cytokine receptor interaction, necroptosis, NOD-like receptor signaling pathway, and apoptosis. The necroptosis and cytokine–cytokine receptor interaction signaling pathways may be responsible for the pathological alterations observed in this study and may help provide insights for the formulation of a strategy for therapeutic intervention, following further investigation. The necroptosis signaling pathway is a programmed form of necrosis. It can be initiated by different stimuli, such as tumor necrosis factor (TNF) and TNF-related apoptosis-inducing ligand. It has previously been demonstrated that cytokine and immune response pathways, transforming growth factor, nitric oxide signaling, and hypertrophic PI3K/AKT signaling pathways are shared among all pBOO phenotypes. AP-1 and NF-kB are considered the dominant transcription factors, and TNF-α is recognized as the top upstream regulator [23]. However, classic fibrosis-related pathways such as the transforming growth factor beta signaling pathway were not identified in this analysis. Therefore, it may be implied that USCs inhibit the initiation of the pathophysiological cascade of events in the bladder wall via the necroptosis and cytokine–cytokine receptor interaction signaling pathways. Additional research is warranted to verify the exact roles of the identified miRNAs, genes, and signaling pathways in the pathogenesis of bladder remodeling induced by pBOO.

## Conclusions

To the best of our knowledge, this study is the first to identify the protective effects of USCs on bladder function and remodeling in pBOO rats. Treatment with USCs elevated bladder compliance and maximal voiding pressure, reduced end-filling pressure and voided volume, strengthened detrusor muscle contractility, and increased sensitivity to carbachol. These alterations were associated with a reduction in collagen deposition in the extracellular matrix and a reduction in cell apoptosis. Alterations in miRNA and mRNA expression levels in the bladder of pBOO rats with and without USC treatment were observed. Bioinformatics analysis of these alterations may help elucidate and improve our understanding of the molecular mechanisms underlying the effects of USCs in pBOO rats. Both miR-142 and miR-9a may play regulatory roles via anti-inflammatory, anti-apoptotic, and antifibrotic effects. Although the mechanisms by which these effects occur have not been fully elucidated, it is suggested that USCs exert their benefits via necroptosis and cytokine–cytokine receptor interaction signaling pathways.

However, our study has certain limitations. We did not perform a luciferase reporter gene experiment to prove the direct regulatory relationship between a miRNA and its target gene. Furthermore, we did not use miR-142 and miR-9a inhibitors for in vivo experiments because the main purpose of this study was to verify the effect of USCs on the bladder function in pBOO rat and to mine the key miRNAs in that process by using RNA sequencing. In future studies, we will further explore the role and regulatory mechanism of USCs in USC-mediated bladder repair strategies in the setting of obstructive injury.

## Supplementary Information

ESM 1 (DOCX 106 kb)

## Data Availability

All statistical data generated or analysed during this study are included in this published article [and its supplementary information files]. The gene datasets generated and/or analyzed in the current study are available in the GEP repository (accession No. GSE171367).

## Abbreviations

DEG: differentially expressed gene
DEM: differentially expressed miRNA
GO: Gene Ontology
H&E: hematoxylin-eosin
KEGG: Kyoto Encyclopedia of Genes and Genomes
ns: no significance
pBOO: partial bladder outlet obstruction
PBS: phosphate-buffered saline
TNF: tumor necrosis factor
USCs: urine-derived stem cells
