# Supplementary material for: Human Urine-Derived Stem Cells Improve Partial Bladder Outlet Obstruction in Rats: Preliminary Data and microRNA-mRNA Expression Profile
Source: Stem Cell Rev Rep. 2022 Mar 1;18(7):2403–13. doi: 10.1007/s12015-022-10340-0 (PMC9489579; doi:10.1007/s12015-022-10340-0)
Supplement: Supplementary file 1 — (DOCX 106 kb) [file 12015_2022_10340_MOESM1_ESM.docx]

Statistical Data

Content:

1. Urodynamic
   1. Compliance
   2. Maximal voiding pressure
   3. End filling pressure
   4. Residual volume
   5. Volume voided
   6. Bladder compacity
2. TUNEL
   1. Statistical data
3. Assessment of detrusor muscle contractility
   1. Statistical data
4. Urodynamics
   1. Compliance

| Two-way ANOVA | Mean Diff. | 95.00% CI of diff. | Significant? | Summary | Adjusted P Value |
| --- | --- | --- | --- | --- | --- |
| Ctrl |  |  |  |  |  |
| 7W vs. 11W | -0.5567 | -9.726 to 8.612 | No | ns | 0.9985 |
| 7W vs. 15W | -0.97 | -10.14 to 8.199 | No | ns | 0.9923 |
| 7W vs. 19W | -1.5 | -10.67 to 7.669 | No | ns | 0.9727 |
| 11W vs. 15W | -0.4133 | -9.582 to 8.756 | No | ns | 0.9994 |
| 11W vs. 19W | -0.9433 | -10.11 to 8.226 | No | ns | 0.9929 |
| 15W vs. 19W | -0.53 | -9.699 to 8.639 | No | ns | 0.9987 |
|  |  |  |  |  |  |
| pBOO |  |  |  |  |  |
| 7W vs. 11W | 2.148 | -7.021 to 11.32 | No | ns | 0.9256 |
| 7W vs. 15W | 4.425 | -4.744 to 13.59 | No | ns | 0.582 |
| 7W vs. 19W | 8.622 | -0.5473 to 17.79 | No | ns | 0.0726 |
| 11W vs. 15W | 2.277 | -6.892 to 11.45 | No | ns | 0.913 |
| 11W vs. 19W | 6.473 | -2.696 to 15.64 | No | ns | 0.2537 |
| 15W vs. 19W | 4.197 | -4.972 to 13.37 | No | ns | 0.6233 |
|  |  |  |  |  |  |
| pBOO+USCs |  |  |  |  |  |
| 7W vs. 11W | -4.027 | -13.20 to 5.142 | No | ns | 0.6538 |
| 7W vs. 15W | -16.41 | -25.58 to -7.239 | Yes | **** | <0.0001 |
| 7W vs. 19W | -26.57 | -35.74 to -17.40 | Yes | **** | <0.0001 |
| 11W vs. 15W | -12.38 | -21.55 to -3.213 | Yes | ** | 0.0039 |
| 11W vs. 19W | -22.54 | -31.71 to -13.37 | Yes | **** | <0.0001 |
| 15W vs. 19W | -10.16 | -19.33 to -0.9894 | Yes | * | 0.0242 |

| Test details | Mean 1 | Mean 2 | Mean Diff. | SE of diff. | N1 | N2 | q | DF |
| --- | --- | --- | --- | --- | --- | --- | --- | --- |
|  |  |  |  |  |  |  |  |  |
| Ctrl |  |  |  |  |  |  |  |  |
| 7W vs. 11W | 48.57 | 49.13 | -0.5567 | 3.47 | 6 | 6 | 0.2269 | 60 |
| 7W vs. 15W | 48.57 | 49.54 | -0.97 | 3.47 | 6 | 6 | 0.3954 | 60 |
| 7W vs. 19W | 48.57 | 50.07 | -1.5 | 3.47 | 6 | 6 | 0.6114 | 60 |
| 11W vs. 15W | 49.13 | 49.54 | -0.4133 | 3.47 | 6 | 6 | 0.1685 | 60 |
| 11W vs. 19W | 49.13 | 50.07 | -0.9433 | 3.47 | 6 | 6 | 0.3845 | 60 |
| 15W vs. 19W | 49.54 | 50.07 | -0.53 | 3.47 | 6 | 6 | 0.216 | 60 |
|  |  |  |  |  |  |  |  |  |
| pBOO |  |  |  |  |  |  |  |  |
| 7W vs. 11W | 21 | 18.85 | 2.148 | 3.47 | 6 | 6 | 0.8756 | 60 |
| 7W vs. 15W | 21 | 16.58 | 4.425 | 3.47 | 6 | 6 | 1.804 | 60 |
| 7W vs. 19W | 21 | 12.38 | 8.622 | 3.47 | 6 | 6 | 3.514 | 60 |
| 11W vs. 15W | 18.85 | 16.58 | 2.277 | 3.47 | 6 | 6 | 0.9279 | 60 |
| 11W vs. 19W | 18.85 | 12.38 | 6.473 | 3.47 | 6 | 6 | 2.638 | 60 |
| 15W vs. 19W | 16.58 | 12.38 | 4.197 | 3.47 | 6 | 6 | 1.71 | 60 |
|  |  |  |  |  |  |  |  |  |
| pBOO+USCs |  |  |  |  |  |  |  |  |
| 7W vs. 11W | 21.94 | 25.97 | -4.027 | 3.47 | 6 | 6 | 1.641 | 60 |
| 7W vs. 15W | 21.94 | 38.35 | -16.41 | 3.47 | 6 | 6 | 6.688 | 60 |
| 7W vs. 19W | 21.94 | 48.51 | -26.57 | 3.47 | 6 | 6 | 10.83 | 60 |
| 11W vs. 15W | 25.97 | 38.35 | -12.38 | 3.47 | 6 | 6 | 5.047 | 60 |
| 11W vs. 19W | 25.97 | 48.51 | -22.54 | 3.47 | 6 | 6 | 9.187 | 60 |
| 15W vs. 19W | 38.35 | 48.51 | -10.16 | 3.47 | 6 | 6 | 4.14 | 60 |

| Two-way ANOVA | Mean Diff. | 95.00% CI of diff. | Significant? | Summary | Adjusted P Value |
| --- | --- | --- | --- | --- | --- |
|  |  |  |  |  |  |
| 7W |  |  |  |  |  |
| Ctrl vs. pBOO | 27.57 | 19.23 to 35.91 | Yes | **** | <0.0001 |
| Ctrl vs. pBOO+USCs | 26.63 | 18.29 to 34.97 | Yes | **** | <0.0001 |
| pBOO vs. pBOO+USCs | -0.9383 | -9.277 to 7.400 | No | ns | 0.9605 |
|  |  |  |  |  |  |
| 11W |  |  |  |  |  |
| Ctrl vs. pBOO | 30.27 | 21.93 to 38.61 | Yes | **** | <0.0001 |
| Ctrl vs. pBOO+USCs | 23.16 | 14.82 to 31.50 | Yes | **** | <0.0001 |
| pBOO vs. pBOO+USCs | -7.113 | -15.45 to 1.225 | No | ns | 0.1092 |
|  |  |  |  |  |  |
| 15W |  |  |  |  |  |
| Ctrl vs. pBOO | 32.96 | 24.62 to 41.30 | Yes | **** | <0.0001 |
| Ctrl vs. pBOO+USCs | 11.19 | 2.851 to 19.53 | Yes | ** | 0.0057 |
| pBOO vs. pBOO+USCs | -21.77 | -30.11 to -13.43 | Yes | **** | <0.0001 |
|  |  |  |  |  |  |
| 19W |  |  |  |  |  |
| Ctrl vs. pBOO | 37.69 | 29.35 to 46.03 | Yes | **** | <0.0001 |
| Ctrl vs. pBOO+USCs | 1.562 | -6.777 to 9.900 | No | ns | 0.8946 |
| pBOO vs. pBOO+USCs | -36.13 | -44.47 to -27.79 | Yes | **** | <0.0001 |

| Test details | Mean 1 | Mean 2 | Mean Diff. | SE of diff. | N1 | N2 | q | DF |
| --- | --- | --- | --- | --- | --- | --- | --- | --- |
|  |  |  |  |  |  |  |  |  |
| 7W |  |  |  |  |  |  |  |  |
| Ctrl vs. pBOO | 48.57 | 21 | 27.57 | 3.47 | 6 | 6 | 11.24 | 60 |
| Ctrl vs. pBOO+USCs | 48.57 | 21.94 | 26.63 | 3.47 | 6 | 6 | 10.85 | 60 |
| pBOO vs. pBOO+USCs | 21 | 21.94 | -0.9383 | 3.47 | 6 | 6 | 0.3824 | 60 |
|  |  |  |  |  |  |  |  |  |
| 11W |  |  |  |  |  |  |  |  |
| Ctrl vs. pBOO | 49.13 | 18.85 | 30.27 | 3.47 | 6 | 6 | 12.34 | 60 |
| Ctrl vs. pBOO+USCs | 49.13 | 25.97 | 23.16 | 3.47 | 6 | 6 | 9.439 | 60 |
| pBOO vs. pBOO+USCs | 18.85 | 25.97 | -7.113 | 3.47 | 6 | 6 | 2.899 | 60 |
|  |  |  |  |  |  |  |  |  |
| 15W |  |  |  |  |  |  |  |  |
| Ctrl vs. pBOO | 49.54 | 16.58 | 32.96 | 3.47 | 6 | 6 | 13.43 | 60 |
| Ctrl vs. pBOO+USCs | 49.54 | 38.35 | 11.19 | 3.47 | 6 | 6 | 4.561 | 60 |
| pBOO vs. pBOO+USCs | 16.58 | 38.35 | -21.77 | 3.47 | 6 | 6 | 8.874 | 60 |
|  |  |  |  |  |  |  |  |  |
| 19W |  |  |  |  |  |  |  |  |
| Ctrl vs. pBOO | 50.07 | 12.38 | 37.69 | 3.47 | 6 | 6 | 15.36 | 60 |
| Ctrl vs. pBOO+USCs | 50.07 | 48.51 | 1.562 | 3.47 | 6 | 6 | 0.6365 | 60 |
| pBOO vs. pBOO+USCs | 12.38 | 48.51 | -36.13 | 3.47 | 6 | 6 | 14.72 | 60 |

- 1. Maximal voiding pressure

| Two-way ANOVA | Mean Diff. | 95.00% CI of diff. | Significant? | Summary | Adjusted P Value |
| --- | --- | --- | --- | --- | --- |
|  |  |  |  |  |  |
| Ctrl |  |  |  |  |  |
| 7W vs. 11W | 1.215 | -4.884 to 7.314 | No | ns | 0.9524 |
| 7W vs. 15W | -0.3383 | -6.437 to 5.760 | No | ns | 0.9989 |
| 7W vs. 19W | -0.295 | -6.394 to 5.804 | No | ns | 0.9992 |
| 11W vs. 15W | -1.553 | -7.652 to 4.545 | No | ns | 0.9069 |
| 11W vs. 19W | -1.51 | -7.609 to 4.589 | No | ns | 0.9136 |
| 15W vs. 19W | 0.04333 | -6.055 to 6.142 | No | ns | >0.9999 |
|  |  |  |  |  |  |
| pBOO |  |  |  |  |  |
| 7W vs. 11W | 0.02833 | -6.070 to 6.127 | No | ns | >0.9999 |
| 7W vs. 15W | 1.713 | -4.385 to 7.812 | No | ns | 0.8795 |
| 7W vs. 19W | 4.268 | -1.830 to 10.37 | No | ns | 0.2609 |
| 11W vs. 15W | 1.685 | -4.414 to 7.784 | No | ns | 0.8846 |
| 11W vs. 19W | 4.24 | -1.859 to 10.34 | No | ns | 0.2664 |
| 15W vs. 19W | 2.555 | -3.544 to 8.654 | No | ns | 0.6866 |
|  |  |  |  |  |  |
| pBOO+USCs |  |  |  |  |  |
| 7W vs. 11W | -7.165 | -13.26 to -1.066 | Yes | * | 0.015 |
| 7W vs. 15W | -13.45 | -19.55 to -7.351 | Yes | **** | <0.0001 |
| 7W vs. 19W | -16.97 | -23.07 to -10.87 | Yes | **** | <0.0001 |
| 11W vs. 15W | -6.285 | -12.38 to -0.1863 | Yes | * | 0.041 |
| 11W vs. 19W | -9.803 | -15.90 to -3.705 | Yes | *** | 0.0004 |
| 15W vs. 19W | -3.518 | -9.617 to 2.580 | No | ns | 0.4295 |

| Test details | Mean 1 | Mean 2 | Mean Diff. | SE of diff. | N1 | N2 | q | DF |
| --- | --- | --- | --- | --- | --- | --- | --- | --- |
|  |  |  |  |  |  |  |  |  |
| Ctrl |  |  |  |  |  |  |  |  |
| 7W vs. 11W | 50.41 | 49.2 | 1.215 | 2.308 | 6 | 6 | 0.7445 | 60 |
| 7W vs. 15W | 50.41 | 50.75 | -0.3383 | 2.308 | 6 | 6 | 0.2073 | 60 |
| 7W vs. 19W | 50.41 | 50.71 | -0.295 | 2.308 | 6 | 6 | 0.1808 | 60 |
| 11W vs. 15W | 49.2 | 50.75 | -1.553 | 2.308 | 6 | 6 | 0.9518 | 60 |
| 11W vs. 19W | 49.2 | 50.71 | -1.51 | 2.308 | 6 | 6 | 0.9253 | 60 |
| 15W vs. 19W | 50.75 | 50.71 | 0.04333 | 2.308 | 6 | 6 | 0.02655 | 60 |

| Two-way ANOVA | Mean Diff. | 95.00% CI of diff. | Significant? | Summary | Adjusted P Value |
| --- | --- | --- | --- | --- | --- |
|  |  |  |  |  |  |
| 7W |  |  |  |  |  |
| Ctrl vs. pBOO | 26.09 | 20.54 to 31.63 | Yes | **** | <0.0001 |
| Ctrl vs. pBOO+USCs | 25.55 | 20.01 to 31.10 | Yes | **** | <0.0001 |
| pBOO vs. pBOO+USCs | -0.5367 | -6.083 to 5.010 | No | ns | 0.9706 |
|  |  |  |  |  |  |
| 11W |  |  |  |  |  |
| Ctrl vs. pBOO | 24.9 | 19.36 to 30.45 | Yes | **** | <0.0001 |
| Ctrl vs. pBOO+USCs | 17.17 | 11.63 to 22.72 | Yes | **** | <0.0001 |
| pBOO vs. pBOO+USCs | -7.73 | -13.28 to -2.184 | Yes | ** | 0.004 |
|  |  |  |  |  |  |
| 15W |  |  |  |  |  |
| Ctrl vs. pBOO | 28.14 | 22.59 to 33.69 | Yes | **** | <0.0001 |
| Ctrl vs. pBOO+USCs | 12.44 | 6.894 to 17.99 | Yes | **** | <0.0001 |
| pBOO vs. pBOO+USCs | -15.7 | -21.25 to -10.15 | Yes | **** | <0.0001 |
|  |  |  |  |  |  |
| 19W |  |  |  |  |  |
| Ctrl vs. pBOO | 30.65 | 25.11 to 36.20 | Yes | **** | <0.0001 |
| Ctrl vs. pBOO+USCs | 8.878 | 3.332 to 14.42 | Yes | *** | 0.0008 |
| pBOO vs. pBOO+USCs | -21.77 | -27.32 to -16.23 | Yes | **** | <0.0001 |

| Test details | Mean 1 | Mean 2 | Mean Diff. | SE of diff. | N1 | N2 | q | DF |
| --- | --- | --- | --- | --- | --- | --- | --- | --- |
|  |  |  |  |  |  |  |  |  |
| 7W |  |  |  |  |  |  |  |  |
| Ctrl vs. pBOO | 50.41 | 24.32 | 26.09 | 2.308 | 6 | 6 | 15.99 | 60 |
| Ctrl vs. pBOO+USCs | 50.41 | 24.86 | 25.55 | 2.308 | 6 | 6 | 15.66 | 60 |
| pBOO vs. pBOO+USCs | 24.32 | 24.86 | -0.5367 | 2.308 | 6 | 6 | 0.3288 | 60 |
|  |  |  |  |  |  |  |  |  |
| 11W |  |  |  |  |  |  |  |  |
| Ctrl vs. pBOO | 49.2 | 24.29 | 24.9 | 2.308 | 6 | 6 | 15.26 | 60 |
| Ctrl vs. pBOO+USCs | 49.2 | 32.02 | 17.17 | 2.308 | 6 | 6 | 10.52 | 60 |
| pBOO vs. pBOO+USCs | 24.29 | 32.02 | -7.73 | 2.308 | 6 | 6 | 4.737 | 60 |
|  |  |  |  |  |  |  |  |  |
| 15W |  |  |  |  |  |  |  |  |
| Ctrl vs. pBOO | 50.75 | 22.61 | 28.14 | 2.308 | 6 | 6 | 17.24 | 60 |
| Ctrl vs. pBOO+USCs | 50.75 | 38.31 | 12.44 | 2.308 | 6 | 6 | 7.623 | 60 |
| pBOO vs. pBOO+USCs | 22.61 | 38.31 | -15.7 | 2.308 | 6 | 6 | 9.62 | 60 |
|  |  |  |  |  |  |  |  |  |
| 19W |  |  |  |  |  |  |  |  |
| Ctrl vs. pBOO | 50.71 | 20.05 | 30.65 | 2.308 | 6 | 6 | 18.78 | 60 |
| Ctrl vs. pBOO+USCs | 50.71 | 41.83 | 8.878 | 2.308 | 6 | 6 | 5.44 | 60 |
| pBOO vs. pBOO+USCs | 20.05 | 41.83 | -21.77 | 2.308 | 6 | 6 | 13.34 | 60 |

- 1. End filling pressure

| Two-way ANOVA | Mean Diff. | 95.00% CI of diff. | Significant? | Summary | Adjusted P Value |
| --- | --- | --- | --- | --- | --- |
|  |  |  |  |  |  |
| Ctrl |  |  |  |  |  |
| 7W vs. 11W | 0.09333 | -3.311 to 3.498 | No | ns | 0.9999 |
| 7W vs. 15W | -0.42 | -3.825 to 2.985 | No | ns | 0.9879 |
| 7W vs. 19W | -0.6833 | -4.088 to 2.721 | No | ns | 0.9514 |
| 11W vs. 15W | -0.5133 | -3.918 to 2.891 | No | ns | 0.9784 |
| 11W vs. 19W | -0.7767 | -4.181 to 2.628 | No | ns | 0.9308 |
| 15W vs. 19W | -0.2633 | -3.668 to 3.141 | No | ns | 0.9969 |
|  |  |  |  |  |  |
| pBOO |  |  |  |  |  |
| 7W vs. 11W | -1.62 | -5.025 to 1.785 | No | ns | 0.5933 |
| 7W vs. 15W | -2.327 | -5.731 to 1.078 | No | ns | 0.2807 |
| 7W vs. 19W | -5.19 | -8.595 to -1.785 | Yes | *** | 0.0009 |
| 11W vs. 15W | -0.7067 | -4.111 to 2.698 | No | ns | 0.9466 |
| 11W vs. 19W | -3.57 | -6.975 to -0.1654 | Yes | * | 0.0364 |
| 15W vs. 19W | -2.863 | -6.268 to 0.5413 | No | ns | 0.1289 |
|  |  |  |  |  |  |
| pBOO+USCs |  |  |  |  |  |
| 7W vs. 11W | -0.025 | -3.430 to 3.380 | No | ns | >0.9999 |
| 7W vs. 15W | 1.42 | -1.985 to 4.825 | No | ns | 0.6896 |
| 7W vs. 19W | 5.01 | 1.605 to 8.415 | Yes | ** | 0.0014 |
| 11W vs. 15W | 1.445 | -1.960 to 4.850 | No | ns | 0.6778 |
| 11W vs. 19W | 5.035 | 1.630 to 8.440 | Yes | ** | 0.0013 |
| 15W vs. 19W | 3.59 | 0.1854 to 6.995 | Yes | * | 0.035 |

| Test details | Mean 1 | Mean 2 | Mean Diff. | SE of diff. | N1 | N2 | q | DF |
| --- | --- | --- | --- | --- | --- | --- | --- | --- |
|  |  |  |  |  |  |  |  |  |
| Ctrl |  |  |  |  |  |  |  |  |
| 7W vs. 11W | 6.835 | 6.742 | 0.09333 | 1.288 | 6 | 6 | 0.1024 | 60 |
| 7W vs. 15W | 6.835 | 7.255 | -0.42 | 1.288 | 6 | 6 | 0.461 | 60 |
| 7W vs. 19W | 6.835 | 7.518 | -0.6833 | 1.288 | 6 | 6 | 0.7501 | 60 |
| 11W vs. 15W | 6.742 | 7.255 | -0.5133 | 1.288 | 6 | 6 | 0.5635 | 60 |
| 11W vs. 19W | 6.742 | 7.518 | -0.7767 | 1.288 | 6 | 6 | 0.8525 | 60 |
| 15W vs. 19W | 7.255 | 7.518 | -0.2633 | 1.288 | 6 | 6 | 0.289 | 60 |
|  |  |  |  |  |  |  |  |  |
| pBOO |  |  |  |  |  |  |  |  |
| 7W vs. 11W | 10.96 | 12.58 | -1.62 | 1.288 | 6 | 6 | 1.778 | 60 |
| 7W vs. 15W | 10.96 | 13.28 | -2.327 | 1.288 | 6 | 6 | 2.554 | 60 |
| 7W vs. 19W | 10.96 | 16.15 | -5.19 | 1.288 | 6 | 6 | 5.697 | 60 |
| 11W vs. 15W | 12.58 | 13.28 | -0.7067 | 1.288 | 6 | 6 | 0.7757 | 60 |
| 11W vs. 19W | 12.58 | 16.15 | -3.57 | 1.288 | 6 | 6 | 3.919 | 60 |
| 15W vs. 19W | 13.28 | 16.15 | -2.863 | 1.288 | 6 | 6 | 3.143 | 60 |

| Two-way ANOVA | Mean Diff. | 95.00% CI of diff. | Significant? | Summary | Adjusted P Value |
| --- | --- | --- | --- | --- | --- |
|  |  |  |  |  |  |
| 7W |  |  |  |  |  |
| Ctrl vs. pBOO | -4.122 | -7.218 to -1.025 | Yes | ** | 0.0062 |
| Ctrl vs. pBOO+USCs | -3.615 | -6.711 to -0.5187 | Yes | * | 0.0183 |
| pBOO vs. pBOO+USCs | 0.5067 | -2.590 to 3.603 | No | ns | 0.9184 |
|  |  |  |  |  |  |
| 11W |  |  |  |  |  |
| Ctrl vs. pBOO | -5.835 | -8.931 to -2.739 | Yes | **** | <0.0001 |
| Ctrl vs. pBOO+USCs | -3.733 | -6.830 to -0.6370 | Yes | * | 0.0143 |
| pBOO vs. pBOO+USCs | 2.102 | -0.9946 to 5.198 | No | ns | 0.2406 |
|  |  |  |  |  |  |
| 15W |  |  |  |  |  |
| Ctrl vs. pBOO | -6.028 | -9.125 to -2.932 | Yes | **** | <0.0001 |
| Ctrl vs. pBOO+USCs | -1.775 | -4.871 to 1.321 | No | ns | 0.3589 |
| pBOO vs. pBOO+USCs | 4.253 | 1.157 to 7.350 | Yes | ** | 0.0046 |
|  |  |  |  |  |  |
| 19W |  |  |  |  |  |
| Ctrl vs. pBOO | -8.628 | -11.72 to -5.532 | Yes | **** | <0.0001 |
| Ctrl vs. pBOO+USCs | 2.078 | -1.018 to 5.175 | No | ns | 0.248 |
| pBOO vs. pBOO+USCs | 10.71 | 7.610 to 13.80 | Yes | **** | <0.0001 |

| Test details | Mean 1 | Mean 2 | Mean Diff. | SE of diff. | N1 | N2 | q | DF |
| --- | --- | --- | --- | --- | --- | --- | --- | --- |
|  |  |  |  |  |  |  |  |  |
| 7W |  |  |  |  |  |  |  |  |
| Ctrl vs. pBOO | 6.835 | 10.96 | -4.122 | 1.288 | 6 | 6 | 4.524 | 60 |
| Ctrl vs. pBOO+USCs | 6.835 | 10.45 | -3.615 | 1.288 | 6 | 6 | 3.968 | 60 |
| pBOO vs. pBOO+USCs | 10.96 | 10.45 | 0.5067 | 1.288 | 6 | 6 | 0.5561 | 60 |
|  |  |  |  |  |  |  |  |  |
| 11W |  |  |  |  |  |  |  |  |
| Ctrl vs. pBOO | 6.742 | 12.58 | -5.835 | 1.288 | 6 | 6 | 6.405 | 60 |
| Ctrl vs. pBOO+USCs | 6.742 | 10.48 | -3.733 | 1.288 | 6 | 6 | 4.098 | 60 |
| pBOO vs. pBOO+USCs | 12.58 | 10.48 | 2.102 | 1.288 | 6 | 6 | 2.307 | 60 |
|  |  |  |  |  |  |  |  |  |
| 15W |  |  |  |  |  |  |  |  |
| Ctrl vs. pBOO | 7.255 | 13.28 | -6.028 | 1.288 | 6 | 6 | 6.617 | 60 |
| Ctrl vs. pBOO+USCs | 7.255 | 9.03 | -1.775 | 1.288 | 6 | 6 | 1.948 | 60 |
| pBOO vs. pBOO+USCs | 13.28 | 9.03 | 4.253 | 1.288 | 6 | 6 | 4.669 | 60 |
|  |  |  |  |  |  |  |  |  |
| 19W |  |  |  |  |  |  |  |  |
| Ctrl vs. pBOO | 7.518 | 16.15 | -8.628 | 1.288 | 6 | 6 | 9.471 | 60 |
| Ctrl vs. pBOO+USCs | 7.518 | 5.44 | 2.078 | 1.288 | 6 | 6 | 2.281 | 60 |
| pBOO vs. pBOO+USCs | 16.15 | 5.44 | 10.71 | 1.288 | 6 | 6 | 11.75 | 60 |

- 1. Residual volume

| Two-way ANOVA | Mean Diff. | 95.00% CI of diff. | Significant? | Summary | Adjusted P Value |
| --- | --- | --- | --- | --- | --- |
|  |  |  |  |  |  |
| Ctrl |  |  |  |  |  |
| 7W vs. 11W | -0.01815 | -0.2642 to 0.2279 | No | ns | 0.9973 |
| 7W vs. 15W | -0.1013 | -0.3474 to 0.1448 | No | ns | 0.6981 |
| 7W vs. 19W | -0.09467 | -0.3408 to 0.1514 | No | ns | 0.7404 |
| 11W vs. 15W | -0.08318 | -0.3293 to 0.1629 | No | ns | 0.8084 |
| 11W vs. 19W | -0.07652 | -0.3226 to 0.1696 | No | ns | 0.844 |
| 15W vs. 19W | 0.006667 | -0.2394 to 0.2528 | No | ns | 0.9999 |
|  |  |  |  |  |  |
| pBOO |  |  |  |  |  |
| 7W vs. 11W | -0.2205 | -0.4666 to 0.02559 | No | ns | 0.0945 |
| 7W vs. 15W | -0.5742 | -0.8203 to -0.3281 | Yes | **** | <0.0001 |
| 7W vs. 19W | -1.146 | -1.392 to -0.8999 | Yes | **** | <0.0001 |
| 11W vs. 15W | -0.3537 | -0.5998 to -0.1076 | Yes | ** | 0.0019 |
| 11W vs. 19W | -0.9255 | -1.172 to -0.6794 | Yes | **** | <0.0001 |
| 15W vs. 19W | -0.5718 | -0.8179 to -0.3257 | Yes | **** | <0.0001 |
|  |  |  |  |  |  |
| pBOO+USCs |  |  |  |  |  |
| 7W vs. 11W | 0.05167 | -0.1944 to 0.2978 | No | ns | 0.9449 |
| 7W vs. 15W | 0.1547 | -0.09142 to 0.4008 | No | ns | 0.3532 |
| 7W vs. 19W | 0.3298 | 0.08375 to 0.5759 | Yes | ** | 0.0042 |
| 11W vs. 15W | 0.103 | -0.1431 to 0.3491 | No | ns | 0.6872 |
| 11W vs. 19W | 0.2782 | 0.03208 to 0.5243 | Yes | * | 0.0207 |
| 15W vs. 19W | 0.1752 | -0.07092 to 0.4213 | No | ns | 0.2471 |

| Test details | Mean 1 | Mean 2 | Mean Diff. | SE of diff. | N1 | N2 | q | DF |
| --- | --- | --- | --- | --- | --- | --- | --- | --- |
|  |  |  |  |  |  |  |  |  |
| Ctrl |  |  |  |  |  |  |  |  |
| 7W vs. 11W | 0.7048 | 0.723 | -0.01815 | 0.09313 | 6 | 6 | 0.2756 | 60 |
| 7W vs. 15W | 0.7048 | 0.8062 | -0.1013 | 0.09313 | 6 | 6 | 1.539 | 60 |
| 7W vs. 19W | 0.7048 | 0.7995 | -0.09467 | 0.09313 | 6 | 6 | 1.438 | 60 |
| 11W vs. 15W | 0.723 | 0.8062 | -0.08318 | 0.09313 | 6 | 6 | 1.263 | 60 |
| 11W vs. 19W | 0.723 | 0.7995 | -0.07652 | 0.09313 | 6 | 6 | 1.162 | 60 |
| 15W vs. 19W | 0.8062 | 0.7995 | 0.006667 | 0.09313 | 6 | 6 | 0.1012 | 60 |
|  |  |  |  |  |  |  |  |  |
| pBOO |  |  |  |  |  |  |  |  |
| 7W vs. 11W | 1.356 | 1.577 | -0.2205 | 0.09313 | 6 | 6 | 3.349 | 60 |
| 7W vs. 15W | 1.356 | 1.93 | -0.5742 | 0.09313 | 6 | 6 | 8.719 | 60 |
| 7W vs. 19W | 1.356 | 2.502 | -1.146 | 0.09313 | 6 | 6 | 17.4 | 60 |
| 11W vs. 15W | 1.577 | 1.93 | -0.3537 | 0.09313 | 6 | 6 | 5.371 | 60 |
| 11W vs. 19W | 1.577 | 2.502 | -0.9255 | 0.09313 | 6 | 6 | 14.05 | 60 |
| 15W vs. 19W | 1.93 | 2.502 | -0.5718 | 0.09313 | 6 | 6 | 8.684 | 60 |
|  |  |  |  |  |  |  |  |  |
| pBOO+USCs |  |  |  |  |  |  |  |  |
| 7W vs. 11W | 1.417 | 1.365 | 0.05167 | 0.09313 | 6 | 6 | 0.7846 | 60 |
| 7W vs. 15W | 1.417 | 1.262 | 0.1547 | 0.09313 | 6 | 6 | 2.349 | 60 |
| 7W vs. 19W | 1.417 | 1.087 | 0.3298 | 0.09313 | 6 | 6 | 5.009 | 60 |
| 11W vs. 15W | 1.365 | 1.262 | 0.103 | 0.09313 | 6 | 6 | 1.564 | 60 |
| 11W vs. 19W | 1.365 | 1.087 | 0.2782 | 0.09313 | 6 | 6 | 4.224 | 60 |
| 15W vs. 19W | 1.262 | 1.087 | 0.1752 | 0.09313 | 6 | 6 | 2.66 | 60 |

| Two-way ANOVA | Mean Diff. | 95.00% CI of diff. | Significant? | Summary | Adjusted P Value |
| --- | --- | --- | --- | --- | --- |
|  |  |  |  |  |  |
| 7W |  |  |  |  |  |
| Ctrl vs. pBOO | -0.6512 | -0.8750 to -0.4274 | Yes | **** | <0.0001 |
| Ctrl vs. pBOO+USCs | -0.7118 | -0.9356 to -0.4880 | Yes | **** | <0.0001 |
| pBOO vs. pBOO+USCs | -0.06067 | -0.2845 to 0.1631 | No | ns | 0.7923 |
|  |  |  |  |  |  |
| 11W |  |  |  |  |  |
| Ctrl vs. pBOO | -0.8535 | -1.077 to -0.6297 | Yes | **** | <0.0001 |
| Ctrl vs. pBOO+USCs | -0.642 | -0.8658 to -0.4182 | Yes | **** | <0.0001 |
| pBOO vs. pBOO+USCs | 0.2115 | -0.01230 to 0.4353 | No | ns | 0.0678 |
|  |  |  |  |  |  |
| 15W |  |  |  |  |  |
| Ctrl vs. pBOO | -1.124 | -1.348 to -0.9002 | Yes | **** | <0.0001 |
| Ctrl vs. pBOO+USCs | -0.4558 | -0.6796 to -0.2320 | Yes | **** | <0.0001 |
| pBOO vs. pBOO+USCs | 0.6682 | 0.4444 to 0.8920 | Yes | **** | <0.0001 |
|  |  |  |  |  |  |
| 19W |  |  |  |  |  |
| Ctrl vs. pBOO | -1.703 | -1.926 to -1.479 | Yes | **** | <0.0001 |
| Ctrl vs. pBOO+USCs | -0.2873 | -0.5111 to -0.06353 | Yes | ** | 0.0085 |
| pBOO vs. pBOO+USCs | 1.415 | 1.191 to 1.639 | Yes | **** | <0.0001 |

| Test details | Mean 1 | Mean 2 | Mean Diff. | SE of diff. | N1 | N2 | q | DF |
| --- | --- | --- | --- | --- | --- | --- | --- | --- |
|  |  |  |  |  |  |  |  |  |
| 7W |  |  |  |  |  |  |  |  |
| Ctrl vs. pBOO | 0.7048 | 1.356 | -0.6512 | 0.09313 | 6 | 6 | 9.889 | 60 |
| Ctrl vs. pBOO+USCs | 0.7048 | 1.417 | -0.7118 | 0.09313 | 6 | 6 | 10.81 | 60 |
| pBOO vs. pBOO+USCs | 1.356 | 1.417 | -0.06067 | 0.09313 | 6 | 6 | 0.9213 | 60 |
|  |  |  |  |  |  |  |  |  |
| 11W |  |  |  |  |  |  |  |  |
| Ctrl vs. pBOO | 0.723 | 1.577 | -0.8535 | 0.09313 | 6 | 6 | 12.96 | 60 |
| Ctrl vs. pBOO+USCs | 0.723 | 1.365 | -0.642 | 0.09313 | 6 | 6 | 9.75 | 60 |
| pBOO vs. pBOO+USCs | 1.577 | 1.365 | 0.2115 | 0.09313 | 6 | 6 | 3.212 | 60 |
|  |  |  |  |  |  |  |  |  |
| 15W |  |  |  |  |  |  |  |  |
| Ctrl vs. pBOO | 0.8062 | 1.93 | -1.124 | 0.09313 | 6 | 6 | 17.07 | 60 |
| Ctrl vs. pBOO+USCs | 0.8062 | 1.262 | -0.4558 | 0.09313 | 6 | 6 | 6.922 | 60 |
| pBOO vs. pBOO+USCs | 1.93 | 1.262 | 0.6682 | 0.09313 | 6 | 6 | 10.15 | 60 |
|  |  |  |  |  |  |  |  |  |
| 19W |  |  |  |  |  |  |  |  |
| Ctrl vs. pBOO | 0.7995 | 2.502 | -1.703 | 0.09313 | 6 | 6 | 25.85 | 60 |
| Ctrl vs. pBOO+USCs | 0.7995 | 1.087 | -0.2873 | 0.09313 | 6 | 6 | 4.363 | 60 |

- 1. Volume voided

| Two-way ANOVA | Mean Diff. | 95.00% CI of diff. | Significant? | Summary | Adjusted P Value |
| --- | --- | --- | --- | --- | --- |
|  |  |  |  |  |  |
| Ctrl |  |  |  |  |  |
| 7W vs. 11W | 5.167 | -26.72 to 37.06 | No | ns | 0.9734 |
| 7W vs. 15W | 4.5 | -27.39 to 36.39 | No | ns | 0.9821 |
| 7W vs. 19W | 21.33 | -10.56 to 53.22 | No | ns | 0.2988 |
| 11W vs. 15W | -0.6667 | -32.56 to 31.22 | No | ns | >0.9999 |
| 11W vs. 19W | 16.17 | -15.72 to 48.06 | No | ns | 0.5417 |
| 15W vs. 19W | 16.83 | -15.06 to 48.72 | No | ns | 0.5075 |
|  |  |  |  |  |  |
| pBOO |  |  |  |  |  |
| 7W vs. 11W | 10.62 | -21.27 to 42.51 | No | ns | 0.8152 |
| 7W vs. 15W | 27.72 | -4.166 to 59.61 | No | ns | 0.1101 |
| 7W vs. 19W | 43.29 | 11.40 to 75.18 | Yes | ** | 0.0037 |
| 11W vs. 15W | 17.11 | -14.78 to 48.99 | No | ns | 0.4936 |
| 11W vs. 19W | 32.67 | 0.7840 to 64.56 | Yes | * | 0.0426 |
| 15W vs. 19W | 15.57 | -16.32 to 47.46 | No | ns | 0.5728 |
|  |  |  |  |  |  |
| pBOO+USCs |  |  |  |  |  |
| 7W vs. 11W | -29.62 | -61.51 to 2.269 | No | ns | 0.0778 |
| 7W vs. 15W | -66.59 | -98.48 to -34.70 | Yes | **** | <0.0001 |
| 7W vs. 19W | -112.1 | -143.9 to -80.17 | Yes | **** | <0.0001 |
| 11W vs. 15W | -36.97 | -68.86 to -5.082 | Yes | * | 0.0168 |
| 11W vs. 19W | -82.44 | -114.3 to -50.55 | Yes | **** | <0.0001 |
| 15W vs. 19W | -45.47 | -77.35 to -13.58 | Yes | ** | 0.0021 |

| Test details | Mean 1 | Mean 2 | Mean Diff. | SE of diff. | N1 | N2 | q | DF |
| --- | --- | --- | --- | --- | --- | --- | --- | --- |
|  |  |  |  |  |  |  |  |  |
| Ctrl |  |  |  |  |  |  |  |  |
| 7W vs. 11W | 337.8 | 332.7 | 5.167 | 12.07 | 6 | 6 | 0.6055 | 60 |
| 7W vs. 15W | 337.8 | 333.3 | 4.5 | 12.07 | 6 | 6 | 0.5274 | 60 |
| 7W vs. 19W | 337.8 | 316.5 | 21.33 | 12.07 | 6 | 6 | 2.5 | 60 |
| 11W vs. 15W | 332.7 | 333.3 | -0.6667 | 12.07 | 6 | 6 | 0.07813 | 60 |
| 11W vs. 19W | 332.7 | 316.5 | 16.17 | 12.07 | 6 | 6 | 1.895 | 60 |
| 15W vs. 19W | 333.3 | 316.5 | 16.83 | 12.07 | 6 | 6 | 1.973 | 60 |
|  |  |  |  |  |  |  |  |  |
| pBOO |  |  |  |  |  |  |  |  |
| 7W vs. 11W | 238.8 | 228.1 | 10.62 | 12.07 | 6 | 6 | 1.244 | 60 |
| 7W vs. 15W | 238.8 | 211 | 27.72 | 12.07 | 6 | 6 | 3.249 | 60 |
| 7W vs. 19W | 238.8 | 195.5 | 43.29 | 12.07 | 6 | 6 | 5.073 | 60 |
| 11W vs. 15W | 228.1 | 211 | 17.11 | 12.07 | 6 | 6 | 2.005 | 60 |
| 11W vs. 19W | 228.1 | 195.5 | 32.67 | 12.07 | 6 | 6 | 3.829 | 60 |
| 15W vs. 19W | 211 | 195.5 | 15.57 | 12.07 | 6 | 6 | 1.824 | 60 |
|  |  |  |  |  |  |  |  |  |
| pBOO+USCs |  |  |  |  |  |  |  |  |
| 7W vs. 11W | 228.7 | 258.3 | -29.62 | 12.07 | 6 | 6 | 3.471 | 60 |
| 7W vs. 15W | 228.7 | 295.3 | -66.59 | 12.07 | 6 | 6 | 7.804 | 60 |
| 7W vs. 19W | 228.7 | 340.7 | -112.1 | 12.07 | 6 | 6 | 13.13 | 60 |
| 11W vs. 15W | 258.3 | 295.3 | -36.97 | 12.07 | 6 | 6 | 4.333 | 60 |
| 11W vs. 19W | 258.3 | 340.7 | -82.44 | 12.07 | 6 | 6 | 9.661 | 60 |
| 15W vs. 19W | 295.3 | 340.7 | -45.47 | 12.07 | 6 | 6 | 5.328 | 60 |

| Two-way ANOVA | Mean Diff. | 95.00% CI of diff. | Significant? | Summary | Adjusted P Value |
| --- | --- | --- | --- | --- | --- |
|  |  |  |  |  |  |
| 7W |  |  |  |  |  |
| Ctrl vs. pBOO | 99.08 | 70.07 to 128.1 | Yes | **** | <0.0001 |
| Ctrl vs. pBOO+USCs | 109.2 | 80.15 to 138.2 | Yes | **** | <0.0001 |
| pBOO vs. pBOO+USCs | 10.08 | -18.93 to 39.08 | No | ns | 0.683 |
|  |  |  |  |  |  |
| 11W |  |  |  |  |  |
| Ctrl vs. pBOO | 104.5 | 75.53 to 133.5 | Yes | **** | <0.0001 |
| Ctrl vs. pBOO+USCs | 74.36 | 45.36 to 103.4 | Yes | **** | <0.0001 |
| pBOO vs. pBOO+USCs | -30.16 | -59.16 to -1.162 | Yes | * | 0.0397 |
|  |  |  |  |  |  |
| 15W |  |  |  |  |  |
| Ctrl vs. pBOO | 122.3 | 93.30 to 151.3 | Yes | **** | <0.0001 |
| Ctrl vs. pBOO+USCs | 38.06 | 9.057 to 67.06 | Yes | ** | 0.007 |
| pBOO vs. pBOO+USCs | -84.24 | -113.2 to -55.24 | Yes | **** | <0.0001 |
|  |  |  |  |  |  |
| 19W |  |  |  |  |  |
| Ctrl vs. pBOO | 121 | 92.03 to 150.0 | Yes | **** | <0.0001 |
| Ctrl vs. pBOO+USCs | -24.24 | -53.24 to 4.762 | No | ns | 0.1189 |
| pBOO vs. pBOO+USCs | -145.3 | -174.3 to -116.3 | Yes | **** | <0.0001 |

| Test details | Mean 1 | Mean 2 | Mean Diff. | SE of diff. | N1 | N2 | q | DF |
| --- | --- | --- | --- | --- | --- | --- | --- | --- |
|  |  |  |  |  |  |  |  |  |
| 7W |  |  |  |  |  |  |  |  |
| Ctrl vs. pBOO | 337.8 | 238.8 | 99.08 | 12.07 | 6 | 6 | 11.61 | 60 |
| Ctrl vs. pBOO+USCs | 337.8 | 228.7 | 109.2 | 12.07 | 6 | 6 | 12.79 | 60 |
| pBOO vs. pBOO+USCs | 238.8 | 228.7 | 10.08 | 12.07 | 6 | 6 | 1.181 | 60 |
|  |  |  |  |  |  |  |  |  |
| 11W |  |  |  |  |  |  |  |  |
| Ctrl vs. pBOO | 332.7 | 228.1 | 104.5 | 12.07 | 6 | 6 | 12.25 | 60 |
| Ctrl vs. pBOO+USCs | 332.7 | 258.3 | 74.36 | 12.07 | 6 | 6 | 8.715 | 60 |
| pBOO vs. pBOO+USCs | 228.1 | 258.3 | -30.16 | 12.07 | 6 | 6 | 3.535 | 60 |
|  |  |  |  |  |  |  |  |  |
| 15W |  |  |  |  |  |  |  |  |
| Ctrl vs. pBOO | 333.3 | 211 | 122.3 | 12.07 | 6 | 6 | 14.33 | 60 |
| Ctrl vs. pBOO+USCs | 333.3 | 295.3 | 38.06 | 12.07 | 6 | 6 | 4.46 | 60 |
| pBOO vs. pBOO+USCs | 211 | 295.3 | -84.24 | 12.07 | 6 | 6 | 9.872 | 60 |
|  |  |  |  |  |  |  |  |  |
| 19W |  |  |  |  |  |  |  |  |
| Ctrl vs. pBOO | 316.5 | 195.5 | 121 | 12.07 | 6 | 6 | 14.18 | 60 |
| Ctrl vs. pBOO+USCs | 316.5 | 340.7 | -24.24 | 12.07 | 6 | 6 | 2.841 | 60 |
| pBOO vs. pBOO+USCs | 195.5 | 340.7 | -145.3 | 12.07 | 6 | 6 | 17.02 | 60 |

- 1. Bladder compacity

| Two-way ANOVA | Mean Diff. | 95.00% CI of diff. | Significant? | Summary | Adjusted P Value |
| --- | --- | --- | --- | --- | --- |
|  |  |  |  |  |  |
| Ctrl |  |  |  |  |  |
| 7W vs. 11W | -0.01667 | -0.3079 to 0.2746 | No | ns | 0.9988 |
| 7W vs. 15W | -0.055 | -0.3463 to 0.2363 | No | ns | 0.959 |
| 7W vs. 19W | -0.03167 | -0.3229 to 0.2596 | No | ns | 0.9917 |
| 11W vs. 15W | -0.03833 | -0.3296 to 0.2529 | No | ns | 0.9854 |
| 11W vs. 19W | -0.015 | -0.3063 to 0.2763 | No | ns | 0.9991 |
| 15W vs. 19W | 0.02333 | -0.2679 to 0.3146 | No | ns | 0.9966 |
|  |  |  |  |  |  |
| pBOO |  |  |  |  |  |
| 7W vs. 11W | -0.2867 | -0.5779 to 0.004585 | No | ns | 0.0553 |
| 7W vs. 15W | -0.6267 | -0.9179 to -0.3354 | Yes | **** | <0.0001 |
| 7W vs. 19W | -1.022 | -1.313 to -0.7304 | Yes | **** | <0.0001 |
| 11W vs. 15W | -0.34 | -0.6313 to -0.04875 | Yes | * | 0.0159 |
| 11W vs. 19W | -0.735 | -1.026 to -0.4437 | Yes | **** | <0.0001 |
| 15W vs. 19W | -0.395 | -0.6863 to -0.1037 | Yes | ** | 0.0037 |
|  |  |  |  |  |  |
| pBOO+USCs |  |  |  |  |  |
| 7W vs. 11W | -0.08167 | -0.3729 to 0.2096 | No | ns | 0.8801 |
| 7W vs. 15W | 0.04 | -0.2513 to 0.3313 | No | ns | 0.9835 |
| 7W vs. 19W | 0.1183 | -0.1729 to 0.4096 | No | ns | 0.7068 |
| 11W vs. 15W | 0.1217 | -0.1696 to 0.4129 | No | ns | 0.6886 |
| 11W vs. 19W | 0.2 | -0.09125 to 0.4913 | No | ns | 0.2767 |
| 15W vs. 19W | 0.07833 | -0.2129 to 0.3696 | No | ns | 0.8925 |

| Test details | Mean 1 | Mean 2 | Mean Diff. | SE of diff. | N1 | N2 | q | DF |
| --- | --- | --- | --- | --- | --- | --- | --- | --- |
|  |  |  |  |  |  |  |  |  |
| Ctrl |  |  |  |  |  |  |  |  |
| 7W vs. 11W | 1.043 | 1.06 | -0.01667 | 0.1102 | 6 | 6 | 0.2139 | 60 |
| 7W vs. 15W | 1.043 | 1.098 | -0.055 | 0.1102 | 6 | 6 | 0.7057 | 60 |
| 7W vs. 19W | 1.043 | 1.075 | -0.03167 | 0.1102 | 6 | 6 | 0.4063 | 60 |
| 11W vs. 15W | 1.06 | 1.098 | -0.03833 | 0.1102 | 6 | 6 | 0.4919 | 60 |
| 11W vs. 19W | 1.06 | 1.075 | -0.015 | 0.1102 | 6 | 6 | 0.1925 | 60 |
| 15W vs. 19W | 1.098 | 1.075 | 0.02333 | 0.1102 | 6 | 6 | 0.2994 | 60 |
|  |  |  |  |  |  |  |  |  |
| pBOO |  |  |  |  |  |  |  |  |
| 7W vs. 11W | 1.543 | 1.83 | -0.2867 | 0.1102 | 6 | 6 | 3.678 | 60 |
| 7W vs. 15W | 1.543 | 2.17 | -0.6267 | 0.1102 | 6 | 6 | 8.041 | 60 |
| 7W vs. 19W | 1.543 | 2.565 | -1.022 | 0.1102 | 6 | 6 | 13.11 | 60 |
| 11W vs. 15W | 1.83 | 2.17 | -0.34 | 0.1102 | 6 | 6 | 4.363 | 60 |
| 11W vs. 19W | 1.83 | 2.565 | -0.735 | 0.1102 | 6 | 6 | 9.431 | 60 |
| 15W vs. 19W | 2.17 | 2.565 | -0.395 | 0.1102 | 6 | 6 | 5.068 | 60 |
|  |  |  |  |  |  |  |  |  |
| pBOO+USCs |  |  |  |  |  |  |  |  |
| 7W vs. 11W | 1.633 | 1.715 | -0.08167 | 0.1102 | 6 | 6 | 1.048 | 60 |
| 7W vs. 15W | 1.633 | 1.593 | 0.04 | 0.1102 | 6 | 6 | 0.5132 | 60 |
| 7W vs. 19W | 1.633 | 1.515 | 0.1183 | 0.1102 | 6 | 6 | 1.518 | 60 |
| 11W vs. 15W | 1.715 | 1.593 | 0.1217 | 0.1102 | 6 | 6 | 1.561 | 60 |
| 11W vs. 19W | 1.715 | 1.515 | 0.2 | 0.1102 | 6 | 6 | 2.566 | 60 |
| 15W vs. 19W | 1.593 | 1.515 | 0.07833 | 0.1102 | 6 | 6 | 1.005 | 60 |

| Two-way ANOVA | Mean Diff. | 95.00% CI of diff. | Significant? | Summary | Adjusted P Value |
| --- | --- | --- | --- | --- | --- |
|  |  |  |  |  |  |
| 7W |  |  |  |  |  |
| Ctrl vs. pBOO | -0.5 | -0.7649 to -0.2351 | Yes | **** | <0.0001 |
| Ctrl vs. pBOO+USCs | -0.59 | -0.8549 to -0.3251 | Yes | **** | <0.0001 |
| pBOO vs. pBOO+USCs | -0.09 | -0.3549 to 0.1749 | No | ns | 0.6943 |
|  |  |  |  |  |  |
| 11W |  |  |  |  |  |
| Ctrl vs. pBOO | -0.77 | -1.035 to -0.5051 | Yes | **** | <0.0001 |
| Ctrl vs. pBOO+USCs | -0.655 | -0.9199 to -0.3901 | Yes | **** | <0.0001 |
| pBOO vs. pBOO+USCs | 0.115 | -0.1499 to 0.3799 | No | ns | 0.5527 |
|  |  |  |  |  |  |
| 15W |  |  |  |  |  |
| Ctrl vs. pBOO | -1.072 | -1.337 to -0.8068 | Yes | **** | <0.0001 |
| Ctrl vs. pBOO+USCs | -0.495 | -0.7599 to -0.2301 | Yes | **** | <0.0001 |
| pBOO vs. pBOO+USCs | 0.5767 | 0.3118 to 0.8415 | Yes | **** | <0.0001 |
|  |  |  |  |  |  |
| 19W |  |  |  |  |  |
| Ctrl vs. pBOO | -1.49 | -1.755 to -1.225 | Yes | **** | <0.0001 |
| Ctrl vs. pBOO+USCs | -0.44 | -0.7049 to -0.1751 | Yes | *** | 0.0005 |
| pBOO vs. pBOO+USCs | 1.05 | 0.7851 to 1.315 | Yes | **** | <0.0001 |

| Test details | Mean 1 | Mean 2 | Mean Diff. | SE of diff. | N1 | N2 | q | DF |
| --- | --- | --- | --- | --- | --- | --- | --- | --- |
|  |  |  |  |  |  |  |  |  |
| 7W |  |  |  |  |  |  |  |  |
| Ctrl vs. pBOO | 1.043 | 1.543 | -0.5 | 0.1102 | 6 | 6 | 6.416 | 60 |
| Ctrl vs. pBOO+USCs | 1.043 | 1.633 | -0.59 | 0.1102 | 6 | 6 | 7.57 | 60 |
| pBOO vs. pBOO+USCs | 1.543 | 1.633 | -0.09 | 0.1102 | 6 | 6 | 1.155 | 60 |
|  |  |  |  |  |  |  |  |  |
| 11W |  |  |  |  |  |  |  |  |
| Ctrl vs. pBOO | 1.06 | 1.83 | -0.77 | 0.1102 | 6 | 6 | 9.88 | 60 |
| Ctrl vs. pBOO+USCs | 1.06 | 1.715 | -0.655 | 0.1102 | 6 | 6 | 8.404 | 60 |
| pBOO vs. pBOO+USCs | 1.83 | 1.715 | 0.115 | 0.1102 | 6 | 6 | 1.476 | 60 |
|  |  |  |  |  |  |  |  |  |
| 15W |  |  |  |  |  |  |  |  |
| Ctrl vs. pBOO | 1.098 | 2.17 | -1.072 | 0.1102 | 6 | 6 | 13.75 | 60 |
| Ctrl vs. pBOO+USCs | 1.098 | 1.593 | -0.495 | 0.1102 | 6 | 6 | 6.351 | 60 |
| pBOO vs. pBOO+USCs | 2.17 | 1.593 | 0.5767 | 0.1102 | 6 | 6 | 7.399 | 60 |
|  |  |  |  |  |  |  |  |  |
| 19W |  |  |  |  |  |  |  |  |
| Ctrl vs. pBOO | 1.075 | 2.565 | -1.49 | 0.1102 | 6 | 6 | 19.12 | 60 |
| Ctrl vs. pBOO+USCs | 1.075 | 1.515 | -0.44 | 0.1102 | 6 | 6 | 5.646 | 60 |
| pBOO vs. pBOO+USCs | 2.565 | 1.515 | 1.05 | 0.1102 | 6 | 6 | 13.47 | 60 |

1. TUNEL
   1. Statistical data

| **Test of Homogeneity of Variances** | | | |
| --- | --- | --- | --- |
|  | | | |
| Levene Statistic | df1 | df2 | Sig. |
| 1.776 | 2 | 15 | .203 |

| **One-way ANOVA** | | | | | |
| --- | --- | --- | --- | --- | --- |
|  | | | | | |
|  | Sum of Squares | df | Mean Square | F | Sig. |
| Between Groups | 6121.444 | 2 | 3060.722 | 153.548 | .000 |
| Within Groups | 299.000 | 15 | 19.933 |  |  |
| Total | 6420.444 | 17 |  |  |  |

**Post Hoc Tests**

| **Multiple Comparisons** | | | | | | |
| --- | --- | --- | --- | --- | --- | --- |
| Dependent Variable: Positive TUNNEL | | | | | | |
|  | (I) group | (J) group | Mean Difference (I-J) | Std. Error | Sig. | 95% Confidence Interval |
|  |  |  |  |  |  | Lower Bound |
| Tamhane | control | pBOO | -44.66667^*^ | 2.76687 | .000 | -53.0185 |
|  |  | pBOO+USCs | -28.16667^*^ | 2.03443 | .000 | -34.0008 |
|  | pBOO | control | 44.66667^*^ | 2.76687 | .000 | 36.3149 |
|  |  | pBOO+USCs | 16.50000^*^ | 2.85287 | .001 | 8.0359 |
|  | pBOO+USCs | control | 28.16667^*^ | 2.03443 | .000 | 22.3325 |
|  |  | pBOO | -16.50000^*^ | 2.85287 | .001 | -24.9641 |

1. Assessment of detrusor muscle contractility
   1. Statistical data

| Two-way ANOVA | Mean Diff. | 95.00% CI of diff. | Significant? | Summary | Adjusted P Value |
| --- | --- | --- | --- | --- | --- |
|  |  |  |  |  |  |
| Ctrl |  |  |  |  |  |
| -9 vs. -8 | -0.22 | -0.8163 to 0.3763 | No | ns | 0.8903 |
| -9 vs. -7 | -0.5733 | -1.170 to 0.02298 | No | ns | 0.0667 |
| -9 vs. -6 | -1.035 | -1.631 to -0.4387 | Yes | **** | <0.0001 |
| -9 vs. -5 | -4.54 | -5.136 to -3.944 | Yes | **** | <0.0001 |
| -9 vs. -4 | -4.842 | -5.438 to -4.245 | Yes | **** | <0.0001 |
| -8 vs. -7 | -0.3533 | -0.9496 to 0.2430 | No | ns | 0.5189 |
| -8 vs. -6 | -0.815 | -1.411 to -0.2187 | Yes | ** | 0.0019 |
| -8 vs. -5 | -4.32 | -4.916 to -3.724 | Yes | **** | <0.0001 |
| -8 vs. -4 | -4.622 | -5.218 to -4.025 | Yes | **** | <0.0001 |
| -7 vs. -6 | -0.4617 | -1.058 to 0.1346 | No | ns | 0.2239 |
| -7 vs. -5 | -3.967 | -4.563 to -3.370 | Yes | **** | <0.0001 |
| -7 vs. -4 | -4.268 | -4.865 to -3.672 | Yes | **** | <0.0001 |
| -6 vs. -5 | -3.505 | -4.101 to -2.909 | Yes | **** | <0.0001 |
| -6 vs. -4 | -3.807 | -4.403 to -3.210 | Yes | **** | <0.0001 |
| -5 vs. -4 | -0.3017 | -0.8980 to 0.2946 | No | ns | 0.6821 |
|  |  |  |  |  |  |
| pBOO |  |  |  |  |  |
| -9 vs. -8 | -0.3817 | -0.9780 to 0.2146 | No | ns | 0.431 |
| -9 vs. -7 | -0.525 | -1.121 to 0.07131 | No | ns | 0.1172 |
| -9 vs. -6 | -0.7517 | -1.348 to -0.1554 | Yes | ** | 0.0053 |
| -9 vs. -5 | -1.552 | -2.148 to -0.9554 | Yes | **** | <0.0001 |
| -9 vs. -4 | -1.778 | -2.375 to -1.182 | Yes | **** | <0.0001 |
| -8 vs. -7 | -0.1433 | -0.7396 to 0.4530 | No | ns | 0.9815 |
| -8 vs. -6 | -0.37 | -0.9663 to 0.2263 | No | ns | 0.4666 |
| -8 vs. -5 | -1.17 | -1.766 to -0.5737 | Yes | **** | <0.0001 |
| -8 vs. -4 | -1.397 | -1.993 to -0.8004 | Yes | **** | <0.0001 |
| -7 vs. -6 | -0.2267 | -0.8230 to 0.3696 | No | ns | 0.8773 |
| -7 vs. -5 | -1.027 | -1.623 to -0.4304 | Yes | **** | <0.0001 |
| -7 vs. -4 | -1.253 | -1.850 to -0.6570 | Yes | **** | <0.0001 |
| -6 vs. -5 | -0.8 | -1.396 to -0.2037 | Yes | ** | 0.0024 |
| -6 vs. -4 | -1.027 | -1.623 to -0.4304 | Yes | **** | <0.0001 |
| -5 vs. -4 | -0.2267 | -0.8230 to 0.3696 | No | ns | 0.8773 |
|  |  |  |  |  |  |
| pBOO+USCs |  |  |  |  |  |
| -9 vs. -8 | -0.195 | -0.7913 to 0.4013 | No | ns | 0.9315 |
| -9 vs. -7 | -0.3317 | -0.9280 to 0.2646 | No | ns | 0.5879 |
| -9 vs. -6 | -0.51 | -1.106 to 0.08631 | No | ns | 0.1379 |
| -9 vs. -5 | -2.888 | -3.485 to -2.292 | Yes | **** | <0.0001 |
| -9 vs. -4 | -2.862 | -3.458 to -2.265 | Yes | **** | <0.0001 |
| -8 vs. -7 | -0.1367 | -0.7330 to 0.4596 | No | ns | 0.985 |
| -8 vs. -6 | -0.315 | -0.9113 to 0.2813 | No | ns | 0.6407 |
| -8 vs. -5 | -2.693 | -3.290 to -2.097 | Yes | **** | <0.0001 |
| -8 vs. -4 | -2.667 | -3.263 to -2.070 | Yes | **** | <0.0001 |
| -7 vs. -6 | -0.1783 | -0.7746 to 0.4180 | No | ns | 0.9525 |
| -7 vs. -5 | -2.557 | -3.153 to -1.960 | Yes | **** | <0.0001 |
| -7 vs. -4 | -2.53 | -3.126 to -1.934 | Yes | **** | <0.0001 |
| -6 vs. -5 | -2.378 | -2.975 to -1.782 | Yes | **** | <0.0001 |
| -6 vs. -4 | -2.352 | -2.948 to -1.755 | Yes | **** | <0.0001 |
| -5 vs. -4 | 0.02667 | -0.5696 to 0.6230 | No | ns | >0.9999 |

| Test details | Mean 1 | Mean 2 | Mean Diff. | SE of diff. | N1 | N2 | q | DF |
| --- | --- | --- | --- | --- | --- | --- | --- | --- |
|  |  |  |  |  |  |  |  |  |
| Ctrl |  |  |  |  |  |  |  |  |
| -9 vs. -8 | 1.498 | 1.718 | -0.22 | 0.2048 | 6 | 6 | 1.519 | 90 |
| -9 vs. -7 | 1.498 | 2.072 | -0.5733 | 0.2048 | 6 | 6 | 3.96 | 90 |
| -9 vs. -6 | 1.498 | 2.533 | -1.035 | 0.2048 | 6 | 6 | 7.148 | 90 |
| -9 vs. -5 | 1.498 | 6.038 | -4.54 | 0.2048 | 6 | 6 | 31.35 | 90 |
| -9 vs. -4 | 1.498 | 6.34 | -4.842 | 0.2048 | 6 | 6 | 33.44 | 90 |
| -8 vs. -7 | 1.718 | 2.072 | -0.3533 | 0.2048 | 6 | 6 | 2.44 | 90 |
| -8 vs. -6 | 1.718 | 2.533 | -0.815 | 0.2048 | 6 | 6 | 5.629 | 90 |
| -8 vs. -5 | 1.718 | 6.038 | -4.32 | 0.2048 | 6 | 6 | 29.83 | 90 |
| -8 vs. -4 | 1.718 | 6.34 | -4.622 | 0.2048 | 6 | 6 | 31.92 | 90 |
| -7 vs. -6 | 2.072 | 2.533 | -0.4617 | 0.2048 | 6 | 6 | 3.188 | 90 |
| -7 vs. -5 | 2.072 | 6.038 | -3.967 | 0.2048 | 6 | 6 | 27.39 | 90 |
| -7 vs. -4 | 2.072 | 6.34 | -4.268 | 0.2048 | 6 | 6 | 29.48 | 90 |
| -6 vs. -5 | 2.533 | 6.038 | -3.505 | 0.2048 | 6 | 6 | 24.21 | 90 |
| -6 vs. -4 | 2.533 | 6.34 | -3.807 | 0.2048 | 6 | 6 | 26.29 | 90 |
| -5 vs. -4 | 6.038 | 6.34 | -0.3017 | 0.2048 | 6 | 6 | 2.083 | 90 |
|  |  |  |  |  |  |  |  |  |
| pBOO |  |  |  |  |  |  |  |  |
| -9 vs. -8 | 1.293 | 1.675 | -0.3817 | 0.2048 | 6 | 6 | 2.636 | 90 |
| -9 vs. -7 | 1.293 | 1.818 | -0.525 | 0.2048 | 6 | 6 | 3.626 | 90 |
| -9 vs. -6 | 1.293 | 2.045 | -0.7517 | 0.2048 | 6 | 6 | 5.191 | 90 |
| -9 vs. -5 | 1.293 | 2.845 | -1.552 | 0.2048 | 6 | 6 | 10.72 | 90 |
| -9 vs. -4 | 1.293 | 3.072 | -1.778 | 0.2048 | 6 | 6 | 12.28 | 90 |
| -8 vs. -7 | 1.675 | 1.818 | -0.1433 | 0.2048 | 6 | 6 | 0.9899 | 90 |
| -8 vs. -6 | 1.675 | 2.045 | -0.37 | 0.2048 | 6 | 6 | 2.555 | 90 |
| -8 vs. -5 | 1.675 | 2.845 | -1.17 | 0.2048 | 6 | 6 | 8.08 | 90 |
| -8 vs. -4 | 1.675 | 3.072 | -1.397 | 0.2048 | 6 | 6 | 9.646 | 90 |
| -7 vs. -6 | 1.818 | 2.045 | -0.2267 | 0.2048 | 6 | 6 | 1.565 | 90 |
| -7 vs. -5 | 1.818 | 2.845 | -1.027 | 0.2048 | 6 | 6 | 7.09 | 90 |
| -7 vs. -4 | 1.818 | 3.072 | -1.253 | 0.2048 | 6 | 6 | 8.656 | 90 |
| -6 vs. -5 | 2.045 | 2.845 | -0.8 | 0.2048 | 6 | 6 | 5.525 | 90 |
| -6 vs. -4 | 2.045 | 3.072 | -1.027 | 0.2048 | 6 | 6 | 7.09 | 90 |
| -5 vs. -4 | 2.845 | 3.072 | -0.2267 | 0.2048 | 6 | 6 | 1.565 | 90 |
|  |  |  |  |  |  |  |  |  |
| pBOO+USCs |  |  |  |  |  |  |  |  |
| -9 vs. -8 | 1.688 | 1.883 | -0.195 | 0.2048 | 6 | 6 | 1.347 | 90 |
| -9 vs. -7 | 1.688 | 2.02 | -0.3317 | 0.2048 | 6 | 6 | 2.291 | 90 |
| -9 vs. -6 | 1.688 | 2.198 | -0.51 | 0.2048 | 6 | 6 | 3.522 | 90 |
| -9 vs. -5 | 1.688 | 4.577 | -2.888 | 0.2048 | 6 | 6 | 19.95 | 90 |
| -9 vs. -4 | 1.688 | 4.55 | -2.862 | 0.2048 | 6 | 6 | 19.76 | 90 |
| -8 vs. -7 | 1.883 | 2.02 | -0.1367 | 0.2048 | 6 | 6 | 0.9438 | 90 |
| -8 vs. -6 | 1.883 | 2.198 | -0.315 | 0.2048 | 6 | 6 | 2.175 | 90 |
| -8 vs. -5 | 1.883 | 4.577 | -2.693 | 0.2048 | 6 | 6 | 18.6 | 90 |
| -8 vs. -4 | 1.883 | 4.55 | -2.667 | 0.2048 | 6 | 6 | 18.42 | 90 |
| -7 vs. -6 | 2.02 | 2.198 | -0.1783 | 0.2048 | 6 | 6 | 1.232 | 90 |
| -7 vs. -5 | 2.02 | 4.577 | -2.557 | 0.2048 | 6 | 6 | 17.66 | 90 |
| -7 vs. -4 | 2.02 | 4.55 | -2.53 | 0.2048 | 6 | 6 | 17.47 | 90 |
| -6 vs. -5 | 2.198 | 4.577 | -2.378 | 0.2048 | 6 | 6 | 16.43 | 90 |
| -6 vs. -4 | 2.198 | 4.55 | -2.352 | 0.2048 | 6 | 6 | 16.24 | 90 |
| -5 vs. -4 | 4.577 | 4.55 | 0.02667 | 0.2048 | 6 | 6 | 0.1842 | 90 |

| Two-way ANOVA | Mean Diff. | 95.00% CI of diff. | Significant? | Summary | Adjusted P Value |
| --- | --- | --- | --- | --- | --- |
|  |  |  |  |  |  |
| -9 |  |  |  |  |  |
| Ctrl vs. pBOO | 0.205 | -0.2830 to 0.6930 | No | ns | 0.5781 |
| Ctrl vs. pBOO+USCs | -0.19 | -0.6780 to 0.2980 | No | ns | 0.6242 |
| pBOO vs. pBOO+USCs | -0.395 | -0.8830 to 0.09300 | No | ns | 0.1365 |
|  |  |  |  |  |  |
| -8 |  |  |  |  |  |
| Ctrl vs. pBOO | 0.04333 | -0.4447 to 0.5313 | No | ns | 0.9756 |
| Ctrl vs. pBOO+USCs | -0.165 | -0.6530 to 0.3230 | No | ns | 0.7004 |
| pBOO vs. pBOO+USCs | -0.2083 | -0.6963 to 0.2797 | No | ns | 0.5679 |
|  |  |  |  |  |  |
| -7 |  |  |  |  |  |
| Ctrl vs. pBOO | 0.2533 | -0.2347 to 0.7413 | No | ns | 0.4346 |
| Ctrl vs. pBOO+USCs | 0.05167 | -0.4363 to 0.5397 | No | ns | 0.9655 |
| pBOO vs. pBOO+USCs | -0.2017 | -0.6897 to 0.2863 | No | ns | 0.5883 |
|  |  |  |  |  |  |
| -6 |  |  |  |  |  |
| Ctrl vs. pBOO | 0.4883 | 0.0003339 to 0.9763 | Yes | * | 0.0498 |
| Ctrl vs. pBOO+USCs | 0.335 | -0.1530 to 0.8230 | No | ns | 0.236 |
| pBOO vs. pBOO+USCs | -0.1533 | -0.6413 to 0.3347 | No | ns | 0.7351 |
|  |  |  |  |  |  |
| -5 |  |  |  |  |  |
| Ctrl vs. pBOO | 3.193 | 2.705 to 3.681 | Yes | **** | <0.0001 |
| Ctrl vs. pBOO+USCs | 1.462 | 0.9737 to 1.950 | Yes | **** | <0.0001 |
| pBOO vs. pBOO+USCs | -1.732 | -2.220 to -1.244 | Yes | **** | <0.0001 |
|  |  |  |  |  |  |
| -4 |  |  |  |  |  |
| Ctrl vs. pBOO | 3.268 | 2.780 to 3.756 | Yes | **** | <0.0001 |
| Ctrl vs. pBOO+USCs | 1.79 | 1.302 to 2.278 | Yes | **** | <0.0001 |
| pBOO vs. pBOO+USCs | -1.478 | -1.966 to -0.9903 | Yes | **** | <0.0001 |

| Test details | Mean 1 | Mean 2 | Mean Diff. | SE of diff. | N1 | N2 | q | DF |
| --- | --- | --- | --- | --- | --- | --- | --- | --- |
|  |  |  |  |  |  |  |  |  |
| -9 |  |  |  |  |  |  |  |  |
| Ctrl vs. pBOO | 1.498 | 1.293 | 0.205 | 0.2048 | 6 | 6 | 1.416 | 90 |
| Ctrl vs. pBOO+USCs | 1.498 | 1.688 | -0.19 | 0.2048 | 6 | 6 | 1.312 | 90 |
| pBOO vs. pBOO+USCs | 1.293 | 1.688 | -0.395 | 0.2048 | 6 | 6 | 2.728 | 90 |
|  |  |  |  |  |  |  |  |  |
| -8 |  |  |  |  |  |  |  |  |
| Ctrl vs. pBOO | 1.718 | 1.675 | 0.04333 | 0.2048 | 6 | 6 | 0.2993 | 90 |
| Ctrl vs. pBOO+USCs | 1.718 | 1.883 | -0.165 | 0.2048 | 6 | 6 | 1.14 | 90 |
| pBOO vs. pBOO+USCs | 1.675 | 1.883 | -0.2083 | 0.2048 | 6 | 6 | 1.439 | 90 |
|  |  |  |  |  |  |  |  |  |
| -7 |  |  |  |  |  |  |  |  |
| Ctrl vs. pBOO | 2.072 | 1.818 | 0.2533 | 0.2048 | 6 | 6 | 1.75 | 90 |
| Ctrl vs. pBOO+USCs | 2.072 | 2.02 | 0.05167 | 0.2048 | 6 | 6 | 0.3568 | 90 |
| pBOO vs. pBOO+USCs | 1.818 | 2.02 | -0.2017 | 0.2048 | 6 | 6 | 1.393 | 90 |
|  |  |  |  |  |  |  |  |  |
| -6 |  |  |  |  |  |  |  |  |
| Ctrl vs. pBOO | 2.533 | 2.045 | 0.4883 | 0.2048 | 6 | 6 | 3.373 | 90 |
| Ctrl vs. pBOO+USCs | 2.533 | 2.198 | 0.335 | 0.2048 | 6 | 6 | 2.314 | 90 |
| pBOO vs. pBOO+USCs | 2.045 | 2.198 | -0.1533 | 0.2048 | 6 | 6 | 1.059 | 90 |
|  |  |  |  |  |  |  |  |  |
| -5 |  |  |  |  |  |  |  |  |
| Ctrl vs. pBOO | 6.038 | 2.845 | 3.193 | 0.2048 | 6 | 6 | 22.05 | 90 |
| Ctrl vs. pBOO+USCs | 6.038 | 4.577 | 1.462 | 0.2048 | 6 | 6 | 10.09 | 90 |
| pBOO vs. pBOO+USCs | 2.845 | 4.577 | -1.732 | 0.2048 | 6 | 6 | 11.96 | 90 |
|  |  |  |  |  |  |  |  |  |
| -4 |  |  |  |  |  |  |  |  |
| Ctrl vs. pBOO | 6.34 | 3.072 | 3.268 | 0.2048 | 6 | 6 | 22.57 | 90 |
| Ctrl vs. pBOO+USCs | 6.34 | 4.55 | 1.79 | 0.2048 | 6 | 6 | 12.36 | 90 |
| pBOO vs. pBOO+USCs | 3.072 | 4.55 | -1.478 | 0.2048 | 6 | 6 | 10.21 | 90 |
